# Genomic Regions Associated with Respiratory Disease in Holstein Calves in the Southern United States

**DOI:** 10.3390/genes16070741

**Published:** 2025-06-26

**Authors:** Allison L. Herrick, Jennifer N. Kiser, Stephen N. White, Holly L. Neibergs

**Affiliations:** 1 Department of Animal Sciences, Washington State University, Pullman, WA 99164, USA; allison.herrick@wsu.edu (A.L.H.); jennifer.kiser@wsu.edu (J.N.K.); 2 Washington Animal Disease Diagnostics Laboratory, Pullman, WA 99164, USA; 3 Department of Veterinary Microbiology and Pathology, Washington State University, Pullman, WA 99164, USA; 4 United States Department of Agriculture, Agricultural Research Service, Athens, GA 30605, USA

**Keywords:** bovine respiratory disease, genome wide association analysis, gene set enrichment analysis, cattle

## Abstract

Background/Objectives: Bovine respiratory disease (BRD) is a common disease impacting cattle throughout the US. BRD is a multifactorial disease as disease risk varies with the genetic profile of the host, environmental conditions, and pathogen exposure. Selection for enhanced BRD resistant cattle can aid in reducing BRD. The objectives of this study were to identify loci, gene sets, and genes associated and enriched for BRD in pre- and post-weaned Holstein cattle. Methods: Cases consisted of 2147 and 5607 calves treated for BRD as pre-weaned (0–60 days old) and post-weaned (61–420 days old) calves, respectively. Controls consisted of calves untreated for BRD that remained in the herd for 61 (*n* = 14,219) days for pre-weaned or 421 (*n* = 12,242) days for post-weaned calves. A genome-wide association analysis (GWAA) identified loci and positional candidate genes associated with BRD (uncorrected *P* < 1 × 10^−5^) for additive, dominant, and recessive inheritance models. A gene set enrichment analysis (GSEA-SNP) identified gene sets and leading-edge genes enriched (NES ≥ 3) for BRD. Results: In pre-weaned calves, 62 loci and 123 positional candidate genes were associated (*P* < 1 × 10^−5^) in addition to the 12 gene sets and 126 leading-edge genes enriched (NES ≥ 3) for BRD. In post-weaned calves, 181 loci and 185 positional candidate genes were associated (*P* < 1 × 10^−5^), and 63 gene sets and 849 leading-edge genes were enriched (NES ≥ 3) for BRD. Conclusions: These results provide further insight and validation of genomic regions that enhance selection for BRD resistance and for healthier cattle.

## 1. Introduction

Dairy cattle face many different diseases throughout their lives, and one of the most common is bovine respiratory disease (BRD), which can lead to reduced animal performance throughout the duration of the lives of infected individuals [1]. In calves, respiratory disease is one of the leading causes of morbidity and mortality [2,3]. The presentation of BRD is typically the result of a bacterial and/or viral infection. The most common bacterial pathogens affiliated with BRD are *Mannheimia haemolytica*, *Pasteurella multocida*, *Histophilus somni*, *Mycoplasma bovis*, and *Trueperella pyogenes*, and common viruses associated with BRD are bovine respiratory syncytial virus (BRSV), bovine herpesvirus 1, and bovine viral diarrhea virus (BVDV) [4].

Bovine respiratory disease is multifactorial in nature, making the onset of disease the result of a variety of environmental, management and genetic factors [5]. Rates of BRD can fluctuate based upon ventilation and housing parameters, vaccination and colostrum protocols, and even the specific geographical location of the dairy as this may influence the pathogens the animal is exposed to [6,7]. Ensuring proper environmental management and disease prevention strategies are crucial for minimizing the risk of BRD; however, these factors alone have not significantly reduced the impacts of BRD.

Beyond environmental influences, some individuals possess an increased resistance or susceptibility to BRD due to their genetic make-up [8]. Previous cattle respiratory disease studies have estimated the heritability of BRD traits to fall between 0.04 and 0.28 [9,10,11]. Identifying these genomic regions associated with respiratory disease presents an additional tool for producers to utilize in reducing the impact of BRD. Selection for enhanced disease resistance has been highlighted as an area of importance across the dairy industry, and as a result, a health index was incorporated into the Net Merit Dollars (NM$) index in 2018 for Holsteins [12]. However, this index did not include BRD. In contrast, the Dairy Wellness Profit (DWP$), Wellness Trait (WT$), and Calf Wellness (CW$) indexes from Zoetis do include BRD as a trait within their selection indexes based on a genetic study of BRD treatment records performed by the company [13,14].

Continuing to improve the identification of genomic regions associated with BRD across different regions, management styles, and the age when animals are being affected can identify improved markers to use for selection for enhanced resistance to BRD. Due to these factors, the confirmation or “validation” of BRD loci shared across multiple independent populations is essential to ensure the best markers are being utilized for selection. As such, the objective of this study was to identify loci and positional candidate genes associated with BRD, and gene sets and leading-edge genes enriched for BRD in pre-weaned and post-weaned Holstein calves from a single dairy.

## 2. Materials and Methods

### 2.1. Study Population

Genotypes were obtained from a single Southern dairy, where the 22,305 Holsteins resided. This study (#6743) was approved by the Institutional Animal Care and Use Committee of Washington State University. Phenotypic data were gathered from the farm’s Dairy Comp 305 (Valley Agricultural Software, Tulare, CA, USA) software, and genotypes were obtained from STgenetics (Navasota, TX, USA).

The study encompassed two ages of heifers, a pre-weaned group (from birth to 60 days of age) and a post-weaned group (from 61 to 420 days of age). Pre-weaned animals were housed individually in hutches, while weaned animals were housed in group pens. Animals were grouped into cases and controls for each age group. Cases were individuals with at least one reported BRD treatment within the pre-weaned (*n* = 2147) and post-weaned (*n* = 5607) periods. Controls remained within the herd for 61 days for the pre-weaned age group (*n* = 14,219) and for 420 days for the post-weaned age group (*n* = 12,242) and were never treated for BRD. In the pre-weaned age group, 13% of heifers were treated for BRD, and in the post-weaned age group, 31% of heifers were treated for BRD.

### 2.2. Genotyping

Genotyping was completed using the STgenetics (Navasota, TX, USA) genotyping platform. The genotypes received were converted from AB format to ATCG format using R v. 3.6.3, then imputed to approximately 620,000 SNPs using Beagle 4.0 [15] and the ARS-UCD 1.2 assembly “http:/bovinegenome.org (accessed on 8 January 2024)”. Imputation used a reference population that consisted of roughly 4800 Holsteins from California, Idaho, New Mexico, and Washington that were all genotyped using Illumina (San Diego, CA, USA) BovineHD BeadChips [11,16,17]. Accuracy of imputation was >95%. Imputed genotypes were used for genome-wide association analysis (GWAA) and gene set enrichment analysis using SNP data (GSEA-SNP).

### 2.3. Quality Control

Quality control was performed on the imputed SNPs and heifers prior to analysis. Before beginning quality control filtering, there were 16,366 pre-weaned heifers with 619,181 SNPs and 17,849 post-weaned heifers with 619,181 SNPs. Genotypes were removed if the call rate < 0.9 (*n* = 391 in pre-weaned, *n* = 398 in post-weaned), a minor allele frequency < 0.01 (*n* = 53,803 for pre-weaned, and *n* = 53,487 in post-weaned), or for failing Hardy–Weinberg equilibrium testing *P* < 10^−150^ (*n* = 1614 in pre-weaned, and *n* = 1666 in post-weaned). There were no individuals removed from either age group for having more than 10% of genotypes not being called. At the end of quality control filtering, the pre-weaned group consisted of 16,366 heifers and 563,373 SNPs, and the post-weaned age group consisted of 17,849 heifers and 563,630 SNPs to be used in analyses.

### 2.4. Genome-Wide Association Analysis

A principal component analysis was completed, and a genomic inflation factor (λ_GC_) was calculated to determine if there was population stratification present in pre-weaned or post-weaned heifers [18]. There was clustering by the year of birth identified in pre- and post-weaned heifers and so birth year was included as a covariate in the GWAA. The GWAA were performed within the SNP and Variation Suite (SVS v. 8.1) software (Golden Helix, Bozeman, MT, USA) using an efficient mixed-model association eXpedited (EMMAX) statistical approach and an identity-by-state relationship matrix. An EMMAX statistical approach was defined as *y* = *Χβ* + *Ζu* + *∈*, where y = a *n* × 1 vector of observed phenotypic values, *Χ* = an *n* × *f* matrix for fixed effects, *β* = *f* × 1 vector for the coefficients of fixed effects, *Ζ* = a matrix containing random effects, *u* = a vector of random effects with variants of allele substitutions in the population, and *∈* = residual effects [19]. Within this analysis, the fixed effects included were the birth year and age group, and the random effects included were the SNPs that comprised the genomic relationship matrix.

As the mode of inheritance for BRD has not been confirmed, additive, dominant, and recessive inheritance models were performed, and results from all three models were evaluated for each population. The additive model identifies associations that occur when having two minor alleles (aa) made it twice as likely to impact BRD compared to having no minor alleles (AA), and half as likely to have an impact when having a single minor allele (Aa). Contrary, the dominant model identifies associations when having one or two minor alleles (Aa or aa) tended to have a larger impact on BRD compared to those not containing a minor allele (AA). The recessive model identifies associations that contain both minor alleles (aa) and tended to have an increased impact on BRD compared to having one or no minor allele (Aa or AA). Markers associated with BRD were established using the Wellcome Trust thresholds for uncorrected *p*-values, with *P* < 1 × 10^−5^ providing evidence for moderate association and *P* < 5 × 10^−7^ providing evidence for strong associations [20]. Both moderate (*P* < 1 × 10^−5^) and strongly (*P* < 5 × 10^−7^) associated markers were identified for completeness, noting that strongly associated markers provided stronger evidence of an association with BRD. Linkage disequilibrium was examined for SNPs associated with BRD within a genomic region using SVS to determine locus boundaries. SNPs with a D’ > 0.7 were determined to be within the same locus [21,22]. Heritability estimates were calculated in SVS using AI-REML, which is a matrix of allele substitution marker effects, otherwise known as a genomic best linear unbiased prediction (GBLUP) [23,24].

### 2.5. Positional Candidate Genes

Using the methods of Gabriel et al. [25], the average haplotype size for both populations was determined to be 28,896 bp. Positional candidate genes were identified within one haplotype (±29 kb) from the associated SNP based on the ARS-UCD 1.2 bovine genome assembly (“http:/bovinegenome.org (accessed on 8 January 2024)”).

### 2.6. Gene Set Enrichment Analysis–Single Nucleotide Polymorphism

Imputed SNPs passing quality control from the pre-weaned (*n* = 563,373) and post-weaned (*n* = 563,630) groups were used as gene proxies for the GSEA-SNP. Due to constraints in the computational power required for multiple imputations, a smaller control population was utilized for the GSEA-SNP. All cases from each group were included in the GSEA-SNP, while controls were randomly selected until an equal number of cases and controls were reached. This resulted in 4294 pre-weaned heifers (2147 cases and 2147 controls) and 11,214 post-weaned heifers (5607 cases and 5607 controls).

SNPs were mapped to 21,039 protein-coding genes within the ARS-UCD 1.2 genome assembly. Gene sets were taken from five databases: Bio Carta (217 gene sets; http://www.genecarta.com/ accessed on 6 January 2025); Gene Ontology, or GO (3147 gene sets; http://www.geneontology.com, accessed on 6 January 2025); Kyoto Encyclopedia of Gene and Genomes, or KEGG (186 gene sets; http://www.genome.jp.kegg, accessed on 6 January 2025); Protein Analysis Through Evolutionary Relationships, or PANTHER (165 gene sets; http://www.pantherdb.org, accessed on 6 January 2025); and Reactome, or R (674 gene sets; http://www.reactome.org, accessed on 6 January 2025). SNPs within a haplotype of a gene were considered as gene proxies. The single SNP chosen as the gene proxy was the SNP with the greatest evidence of an association with BRD. Using the *p*-value for each SNP representing a gene, genes were placed in a ranked list based on their evidence for an association with BRD. The SNP with the smallest *p*-value was ranked first, while the largest *p*-value was ranked last. The analysis was performed within R v. 3.6.3, using the GenGen v. 1 package [26]. Each gene set’s enrichment score was calculated using a running sum statistic after 10,000 phenotype-based permutations in GenABEL v. 3.6.3 and normalized to account for the number of genes present within the respective gene sets [27,28]. Gene sets with a normalized enrichment score (NES) ≥ 3.0 were identified as enriched for BRD [29]. The leading-edge genes, the genes that positively contributed to the peak enrichment score, were compiled for each gene set.

## 3. Results

### 3.1. Genome-Wide Association Analysis Results

**Figure 1:**
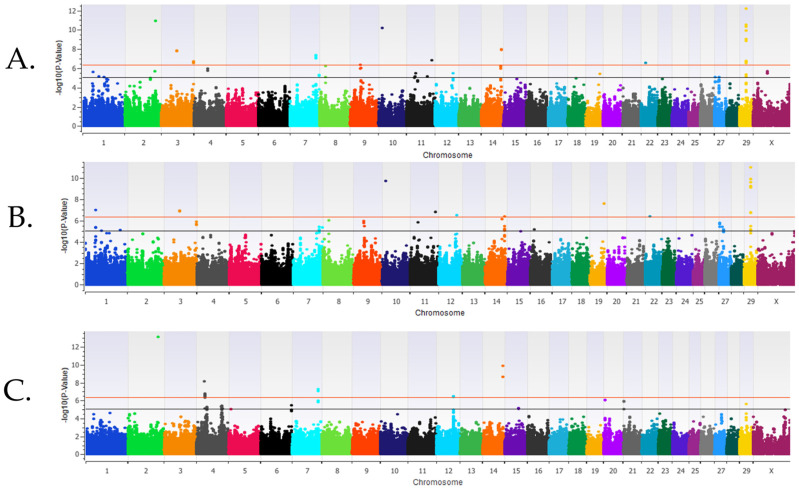
The results for the genome-wide association analysis for bovine respiratory disease are shown for the pre-weaned Holstein heifer calves for the additive inheritance model in (**panel A**), the dominant inheritance model in (**panel B**), and the recessive inheritance model in (**panel C**). Each Manhattan plot has the chromosomes on the x axis and the −log10 *p*-value on the y axis. The significance thresholds for an association with BRD are represented by the lower line (moderate association with an uncorrected *P* < 1 × 10^−5^) and the uppermost line (strong association with an uncorrected *P* < 5 × 10^−7^).

Pre-weaned heifers. The calculated λ_GC_ values for the pre-weaned BRD heifers were 0.98 for the additive model, 0.99 for the dominant model, and 0.95 for the recessive model. Heritability was estimated for BRD in pre-weaned heifers to be 0.06 ± 0.01. The GWAA identified 62 loci and 123 unique positional candidate genes associated with BRD for all inheritance models. Forty positional candidate genes were identified by a BRD-associated SNP being located within an exon or intron of the gene (Supplemental Table S1). Ten loci were strongly associated (*P* < 5 × 10^−7^) with BRD in the additive inheritance model, nine loci were strongly associated with BRD (*P* < 5 × 10^−7^) in the dominant inheritance model, and six loci were strongly associated (*P* < 5 × 10^−7^) with BRD in the recessive model in the pre-weaned calves (Figure 1, Table 1). There were three loci, located on BTA3, BTA22, and BTA29, that were strongly associated (*P* < 5 × 10^−7^) with BRD in both the additive and dominant inheritance models. Another three loci were strongly associated in both the additive and recessive models and were located on BTA2, BTA7, and BTA14.

There were 16 loci moderately associated (*P* < 1 × 10^−5^) with BRD in the additive model, 14 in the dominant model, and 7 in the recessive model for the pre-weaned calves (Supplemental Table S1). There were 40 positional candidate genes with a SNP associated (*P* < 1 × 10^−5^) with BRD in pre-weaned heifers that fell within an exon or intron of a positional candidate gene, and an additional 83 positional candidate genes located within a haplotype of an associated SNP (Supplemental Table S1).

**Figure 2:**
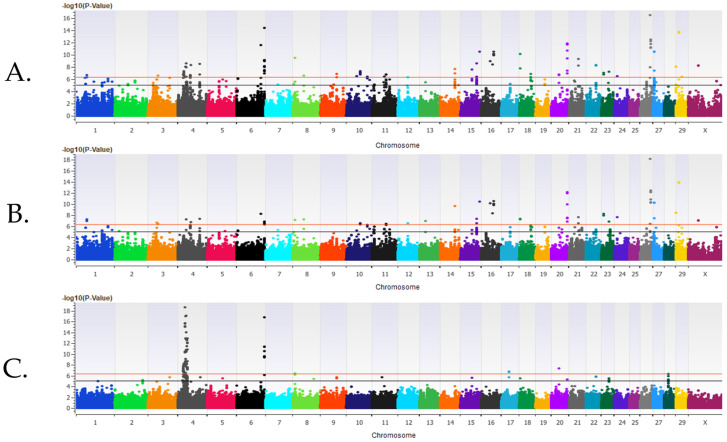
The results for the genome-wide association analysis for bovine respiratory disease are shown for the post-weaned Holstein heifer calves for the additive inheritance model in (**panel A**), the dominant inheritance model in (**panel B**), and the recessive inheritance model in (**panel C**). Each Manhattan plot has the *Bos taurus* chromosomes (BTA) on the x axis and the −log10 *p*-value on the y axis. The significance thresholds for an association with BRD are represented by the lower line (moderate association with an uncorrected *P* < 1 × 10^−5^) and the uppermost line (strong association with an uncorrected *P* < 5 × 10^−7^).

Post-weaned heifers. The λ_GC_ values for the additive, dominant, and recessive inheritance models for BRD in the post-weaned heifers were 0.98, 0.98, and 0.97, respectively. The estimated heritability for BRD was 0.13 ± 0.01 for the post-weaned heifers. There were 181 loci and 185 positional candidate genes associated (*P* < 1 × 10^−5^) with BRD in all inheritance models. Sixty-seven of the positional candidate genes had BRD-associated SNPs located within exons or introns. There were 42 loci strongly associated (*P* < 5 × 10^−7^) with BRD in the additive inheritance model, 33 loci associated with BRD in the dominant inheritance model, and 14 loci associated with BRD in the recessive inheritance model (Figure 2, Table 2). Twenty-nine loci strongly associated (*P* < 5 × 10^−7^) with BRD were shared between the additive and dominant inheritance models and three loci strongly associated (*P* < 5 × 10^−7^) with BRD were shared in the additive and recessive inheritance models.

There were 42 loci moderately (*P* < 1 × 10^−5^) associated with BRD in the additive model, 34 in the dominant inheritance model, and 16 in the recessive model. There were 67 positional candidate genes where the SNP associated (*P* < 1 × 10^−5^) with BRD was within an exon or intron of the gene, and 118 additional positional candidate genes located within one haplotype of the associated SNP.

### 3.2. Gene Set Enrichment Analysis with Single Nucleotide Polymorphisms

Pre-weaned heifers. There were 12 GO gene sets and 126 unique leading-edge genes enriched (NES ≥ 3) for BRD in pre-weaned calves (Supplemental Table S3). Table 3 lists the five gene sets with the largest NES enriched for BRD for the pre-weaned heifers. There were two leading edge genes, polo like kinase 1 (*PLK1*) and menin 1 (*MEN1*), that were shared in half of the gene sets enriched for BRD in pre-weaned heifers (Supplemental Table S3). The 12 enriched gene sets were placed within four different functional categories. The first category, gene sets involved in regulatory functions, included five gene sets, along with the most enriched gene set, negative regulation of protein kinase activity (NES = 3.92) (Supplemental Table S3). Two of the gene sets identified in this gene set group, negative regulation of protein kinase activity and negative regulation of kinase activity, were previously identified as enriched for BRD in beef cattle [30]. The second functional group, cellular differentiation/development, contained four gene sets. The final two functional groups, DNA/RNA regulation and immunity, contained two and a single gene set, respectively.

Post-weaned heifers. There were 63 gene sets and 849 unique leading-edge genes enriched (NES ≥ 3) for BRD in the post-weaned heifers (Supplemental Table S3). Gene sets enriched (NES ≥ 3) for BRD in post-weaned heifers were from GO (*n* = 47), R (*n* = 11), KEGG (*n* = 4), and PANTHER (*n* = 1). The five post-weaned heifer gene sets with the largest NES enriched for BRD are listed in Table 3. In post-weaned heifers, synuclein alpha (*SNCA*) and transforming growth factor beta 1 (*TGFB1*) were also found in nearly half of the gene sets enriched for BRD (Supplemental Table S3). Gene sets enriched (NES ≥ 3) for BRD in pre- and post-weaned heifers can be grouped into nine groups by similarities in their functions (Supplemental Table S3). Twenty-three of the gene sets had functions related to metabolic processes or cellular metabolisms, including the most enriched gene set, purine metabolism (NES = 4.43). The next largest functional group contains 11 gene sets that pertain to cellular responses. There were seven gene sets involved in cellular replication. The remaining seven functional groups contained five or fewer gene sets. Three gene sets (cellular response to oxidative stress, cellular response to oxygen-containing compound, and positive regulation of defense response) enriched for BRD in post-weaned Holstein heifers were previously identified to be associated with BRD in beef cattle (Supplemental Table S5) [30].

### 3.3. Shared Genes and Gene Sets

There were four positional candidate genes (*LOC104968411*, *RAPGEF5*, *TENM3*, and *TFEC*) that were shared between pre- and post-weaned heifers that had BRD-associated SNPs located within an intron or exon, and 12 positional candidate genes identified by SNPs outside of the genes (Table 4). Of the 16 positional candidate genes, transcription factor EC (*TFEC*) was also identified as a leading-edge gene in the post-weaned GSEA-SNP analysis.

There were 36 leading-edge genes that were shared between the pre- and post-weaned heifers (Table 4). *TGFB1* was also one of 36 leading-edge genes that were shared in the pre-weaned and the post-weaned heifer gene sets enriched for BRD (Supplemental Table S3). Further, the shared leading-edge genes, fragile histidine triad diadenosine triphosphatase (*FHIT*), was also identified as a positional candidate gene in post-weaned heifers. The BRD-associated markers located within the intronic regions of *FHIT* were strongly associated (*P* < 5 × 10^−7^) with BRD in the additive inheritance model and moderately associated (*P* < 1 × 10^−5^) within the dominant model in post-weaned heifers.

Comparing the results of this study to previous BRD research allows for the support of these genes and loci as important in BRD susceptibility. There were 633 genes and five gene sets that were identified in the current study that were shared with eight previous studies among different populations and breeds of cattle [10,11,29,30,31,32,33,34]. Of the 633 genes identified across the two current study groups, 66 of them were positional candidate genes, 551 were leading-edge genes, and 16 genes were identified in both analyses (Supplemental Table S4). There was a single gene, *PLCB1*, that was identified in four other BRD studies, and an additional 21 genes that were identified in three other BRD studies. There were also five GO gene sets that were identified in this study that were identified in a previous study in mixed breed beef heifers; two of the gene sets were identified in the pre-weaned BRD population, and three of the gene sets were identified in the post-weaned BRD population (Supplemental Table S5) [30].

## 4. Discussion

### 4.1. Genome-Wide Association Analysis of Pre-Weaned Heifers

In the additive inheritance model, the three loci with the smallest *p*-values were located on BTA2 (*P* = 1.31 × 10^−11^), BTA10 (*P* = 7.48 × 10^−11^), and BTA29 (*P* = 1.72 × 10^−13^) (Supplemental Table S1). There were 13 positional candidate genes located within these three loci, but only one positional candidate gene with a BRD-associated SNP within the gene (intron 4), and it was DENN domain containing 4A (*DENND4A*) on BTA10. *DENND4A* encodes for a member of the DENN protein family, which functions as guanine nucleotide exchange factor (GEFs) for Rab proteins [35]. When activated by GEFs, Rab proteins function within intracellular membranes to assist in receiving cellular signals [36]. Tizioto et al. [34] found *DENND4A* to be differentially expressed in Angus–Hereford crossbreeds that were experimentally challenged with BRSV, BVDV, infectious bovine rhinotracheitis (IBR), and *P. multocida* compared to controls. The results of the current study and the Tizioto et al. [34] study highlight a potentially important gene for selection, as it has been found to be important in BRD in beef and dairy cattle.

In the dominant inheritance model, there were 14 positional candidate genes located in the three loci with the greatest evidence for an association with BRD. These loci were on BTA10 (*P* = 2.02 × 10^−10^), BTA19 (*P* = 2.8 × 10^−8^) and BTA29 (*P* = 1.09 × 10^−11^) (Supplemental Table S1). The loci on BTA10 and BTA29 were also those that had the greatest evidence for an association with BRD in the additive model. There were two positional candidate genes with a BRD-associated SNP within the gene for these three loci. The first positional candidate gene (*DENND4A*) was identified by the same BRD-associated SNP within the fourth intron as in the additive model. The second positional candidate gene (*LOC512869*) was identified by a BRD-associated SNP in intron 25 and was on BTA19. *LOC512869* is ring-finger protein 213-like, and its function is important in the metabolism of lipids and antimicrobial properties [37,38]. *LOC512869’s* role in antimicrobial properties could be important in reducing susceptibility to bacterial BRD pathogens.

The recessive inheritance model had five positional candidate genes located in the three most significant loci. These loci were on BTA2 (*P* = 7.66 × 10^−14^), BTA4 (*P* = 7.66 × 10^−9^), and BTA14 (*P* = 7.66 × 10^−10^) (Supplemental Table S1). None of the three most significant loci were shared with the top three loci from the other two inheritance models in pre-weaned heifers. While there was not a positional candidate gene on BTA2, there was a single positional candidate gene: WW domain containing E3 ubiquitin protein ligase 1 (*WWPI*) on BTA14. *WWP1* is involved in viral internalization and budding in humans by binding to group specific antigen proteins [39]. This process occurs in human T cell leukemia virus type 1, Epstein-Barr virus, and Ebola viruses [40,41]. Whether this process occurs in the viruses common in BRD is unknown, but these viruses have some similarities bovine herpes virus type 1, an element of the BRD complex. *WWP1* is also important in innate immunity responses through the regulation of the DAF-2 insulin-like signaling pathway in *Caenorhabditis. elegans* [42,43]. *WWP1* has previously been identified in two previous BRD studies, where it was found to be differentially expressed in individuals infected with BRSV and IBR, as well as being expressed for BRD traits and immune function [30,34].

The remaining positional candidate genes in the recessive inheritance model for pre-weaned heifers are on BTA4. *LOC104968411*, also known as dynein axonemal heavy chain 11 (*DNAH11*), contains a BRD-associated SNP located within intron 43. *DNAH11* is associated with pregnancy rates, cow and heifer conception rates, and productive life in cattle [44,45]. As BRD impacts reproductive and longevity traits through acute and long-term effects, its association with BRD could partly explain these associations, but further investigation of whether the cattle affected with these reproductive traits were correlated with BRD incidence is needed. In humans, *DNAH11* has a role in primary ciliary dyskinesia, which is a rare condition leading to respiratory complications [46]. In primary ciliary dyskinesia, the respiratory cilia are abnormal, which results in difficulty clearing mucous from the lungs as well as clearing pathogens [47]. *DNAH11* proteins are crucial for proper cilia movement throughout the respiratory tract [48,49]. Thus, *DNAH11* may play a role in BRD by altering mucociliary pathogen clearance in the respiratory tract.

### 4.2. Gene Set Enrichment Analysis—Single Nucleotide Polymorphisms of Pre-Weaned Heifers

The GSEA-SNP identified 126 unique leading-edge genes in 12 gene sets enriched for BRD (Supplemental Table S3). Two leading edge genes, polio-like kinase 1 *(PLK1)* and multiple endocrine neoplasia 1 (*MEN1*), are shared frequently among regulatory gene sets and so will be further discussed. *PLK1* functions in the spatiotemporal regulation of mitosis and meiosis [50]. *PLK1* protein is involved in multiple mitotic events, including spindle entry, the separation of the centrosome, the attachment of microtubules to kinetochores, and cytokinesis [51,52]. Cells that lack *PLK1* expression tend to arrest during the prometaphase portion of mitosis, where centrosomes fail to separate due to deficient microtubule attachment [52]. *PLK1* proteins are important in the functional regulation of the proliferation and contraction of airway muscle cells [53,54]. The control of these muscle cells has been connected to asthma, where the overexpression of *PLK1* was correlated with an increase in asthma and inflammation of pulmonary cells [53]. Though the role of *PLK1’s* in inflammation of respiratory tissues in cattle has yet to be specifically characterized, it has been identified as a leading-edge gene and expressed for BRD in beef cattle [29,30].

*MEN1*, which was also previously identified as a leading-edge gene for feedlot beef cattle with BRD, functions as a tumor suppressor [29,55]. As a tumor suppressor, it has roles in transcription and maintaining genetic integrity in the nucleus [56]. Using knockout mouse models, a reduction of *MEN1* expression has been associated with the onset of pulmonary alveolar proteinosis, which, in humans, is characterized by patients experiencing dyspnea, pulmonary fibrosis, and eventual respiratory failure [57,58]. Mouse models identified that menin assists in regulating macrophage development, chiefly through the reduced expression of granulocyte-macrophage colony stimulating factor [57]. Identifying expression levels of *MEN1* could uncover potential implications of reduced macrophage production or respiratory conditions in cattle.

### 4.3. Genome-Wide Association Analysis of Post-Weaned Heifers

In the additive model, there were 10 positional candidate genes located in the three most significant loci associated with BRD in post-weaned heifers. The most significant loci were located on BTA6 (*P* = 4.6 × 10^−15^), BTA26 (*P* = 3.17 × 10^−17^), and BTA29 (*P* = 1.72 × 10^−14^) (Supplemental Table S2). Of the ten positional candidate genes, only four had BRD-associated SNPs located within an intron or exon of the gene. Actin binding LIM protein family member 2 (*ABLIM2*) on BTA6 was identified as a positional candidate gene with a BRD-associated SNP in intron 12. This positional candidate gene and the same BRD-associated SNP were associated with BRD in the recessive model in post-weaned heifers. In addition to being associated with BRD, *ABLIM2* is associated with increased bleeding risks in humans experiencing pulmonary embolisms and is downregulated in people infected with respiratory syncytial virus [59,60]. It is not known if BRSV, a common BRD pathogen, also downregulates *ABLIM2* expression increasing susceptibility to disease. On BTA26, *LOC100849037* has a BRD-associated SNP in intron seven, and *LOC617705* has a BRD-associated SNP in intron ten. These same BRD-associated SNPs were associated with BRD in the dominant inheritance model for post-weaned heifers. *LOC100849037* is an uncharacterized gene, while *LOC617705* is commonly deleted in brain tumors but has not otherwise been connected with disease traits [61].

The final positional candidate gene in the most significant loci associated with BRD in post-weaned heifers in the additive inheritance model is discs large MAGUK scaffold protein 2 (*DLG2*) on BTA29. Its BRD-associated SNP is within intron 14. *DLG2* is also a positional candidate gene within one of the most significant loci for BRD in the dominant inheritance model and that contains a BRD-associated SNP within the gene. *DLG2* expression is reduced when inflammation is present. The sensing of the inflammation by host cells coupled with a reduced expression of *DLG2* initiates the creation of an inflammasome, a large protein complex that activates pro-inflammatory cytokines that can lead to cell death [62]. Inflammasomes are innate immune system receptors that induce inflammation in response to infectious microbes and molecules [63]. Inflammasomes are important for pathogen mitigation, and under/over activation can lead to increases in infections or chronic inflammation, respectively [63]. Infections related to BRD lead to increased inflammation throughout the pulmonary system, and *DLG2* could play a crucial role in the regulations of inflammation in the lung associated with BRD [64].

In the recessive inheritance model, two (BTA4 and BTA6) of the three most significant BRD associated loci contained positional candidate genes that had a BRD-associated SNP within the gene. There were three positional candidate genes (*KIAA1324L* or *ELAPOR2*, *SEMA3A*, and *SEMA3D*) located within the locus on BTA4 and one positional candidate gene on BTA6, *ABLIM2*, that was discussed previously, which contained a BRD-associated SNP within the gene. The positional candidate gene endosome–lysosome-associated apoptosis and autophagy regulator family member 2 (*KIAA1324L* or *ELAPOR2*) contained a BRD-associated SNP within intron two. In cattle, the *ELAPOR2* protein is upregulated in post-partum cows experiencing an inflammatory response [65]. This is consistent with its role in removing damaged or unnecessary cellular components and maintaining cellular homeostasis through autophagy and apoptosis [66]. Tizioto et al. [34] identified *KIAA1324L* as a differentially expressed gene in the presence of four BRD pathogens, BRSV, BVDV, IBR, and *P. multocida*, suggesting an important role with BRD across breeds, ages, and pathogens. The role of *KIAA1324L* in BRD could also be mediating lung inflammation, autophagy, and apoptosis.

The BRD-associated SNP is in intron 13 in semaphorin 3A (*SEMA3A*) and is in intron 5 for semaphorin 3D (*SEMA3D*). *SEMA3A* was differentially expressed in cattle exposed to *M. haemolytica*, while *SEMA3D* was differentially expressed in cattle infected with BRSV and IBR [34]. *SEMA3A* has been connected to multiple respiratory conditions in humans. Expression of *SEMA3A* inactivates the ERK/JNK pathway, which reduces inflammation during acute respiratory distress syndrome [67]. Acute respiratory distress syndrome is characterized by an accumulation of fluid in the lung that can make breathing difficult and result in organ damage. This condition can be life-threatening if not quickly recognized and treated. *SEMA3A* also recruits T cells, which play an important role in maintaining lung health and protecting against pathogens that infect the lung [68,69]. *SEMA3D* also plays a vital role in the lung in angiogenesis, the formation of airways, and the development of asthma [70,71,72].

### 4.4. Gene Set Enrichment Analysis—Single Nucleotide Polymorphisms in Post-Weaned Heifers

Of the 849 leading-edge genes enriched for BRD in post-weaned heifers, synuclein alpha (*SNCA*) was the most frequently identified, followed by transforming growth factor beta 1 (*TGFB1*). *SNCA* was a leading-edge gene in 31 gene sets, and *TGFB1* was a leading-edge gene in 30 gene sets enriched (NES ≥ 3) for BRD (Supplemental Table S3). *SNCA* is located on BTA6 and results in the proliferation of macrophages, neutrophils, dendritic, and B cells in lung cancers [73]. This is consistent with lower levels of *SNCA* expression correlating with poor lung cancer prognosis. As *SNCA* is active in immune cell infiltration in the lung, the downregulation of *SNCA* would be expected to be detrimental in cattle with BRD. An enrichment of *SNCA* in BRD was previously identified in beef and dairy cattle by Neupane et al. [29], as well as being differentially expressed for beef cattle infected with BVDV, *M. bovis*, *P. multocida*, and *M. haemolytica* [34]. As *SNCA* has been enriched for BRD in multiple studies and is differently expressed when infected with BRD pathogens, this gene should be further investigated into its role in BRD susceptibility.

*TGFB1* is located on BTA18 and is required for the formation of the lung, as well as epithelial–meshenchymal interactions during the development of lung branching and alveolarization [74]. In addition to lung morphogenesis, *TGFB1* has functions regulating immune responses (particularly T cell homeostasis and inflammation), cell growth, and producing extracellular matrices [75]. The upregulation of TGF-β ligands is observed in pulmonary diseases, such as pulmonary fibrosis, emphysema, bronchial asthma, and lung cancer [74]. Denney et al. [76] identified that the production of the *TGFβ* protein suppresses the production and release of interferon beta production in the regulation of cytokines and viral responses. *TGFB1* has previously been identified as an upstream regulator of gene expression in response to BRD pathogen challenges in crossbred Angus cattle and was enriched in a previous BRD pathway analysis [29,34]. The current study confirms the importance of *TGFB1* in susceptibility to BRD.

### 4.5. Sharing of Bovine Respiratory Disease Positional Candidate and Leading-Edge Genes

The sharing of positional candidate genes and leading-edge genes within each population provide important insights into genes that are strongly supported as having a role in BRD. Only one gene in the pre-weaned heifers was shared as a positional candidate and leading-edge gene, whereas there were 11 genes shared in the post-weaned heifers. Suppressor of cytokine signaling 5 (*SOCS5*) was shared as a positional candidate and leading-edge gene for BRD in pre-weaned heifers (Table 4). *SOCS5* is one of four *SOCS* proteins that regulate growth factors, and they are highly expressed in B and T cells, especially in lymphoid organs [77]. *SOCS5*-deficient mice were more likely to become infected with influenza and respond with an increased presence of pro-inflammatory cytokines in pulmonary epithelium [78]. Reduced expression of *SOCS5* is found in chronic obstructive pulmonary disease, while in pulmonary hypertension, the downregulation of *SOCS5* has led to abnormal proliferation and contraction of pulmonary muscle cells [79,80]. Dysregulation of *SOCS5* may also lead to a pulmonary environment conducive to BRD but has yet to be studied.

Two genes, ectonucleoside triphosphate diphosphohydrolase 5 (*ENTPD5)* and NME/NM23 family member 7 (*NME7*), were the most frequently shared genes in the post-weaned heifers, as they were enriched for BRD in over 20 of the gene sets and had BRD-associated SNPs within intronic regions of the gene (Table 4). *ENTPD5* on BTA10 was moderately associated (*P* < 1 × 10^−5^) with BRD in both the additive and dominant inheritance models and was a leading-edge gene in 24 gene sets. The BRD-enriched SNP for this locus was located within the first intron of the gene. *ENTPD5* is expressed as an enzyme within the endoplasmic reticulum, where it assists in its folding [81]. *ENTPD5* has also previously been identified as enriched with BRD in pre-weaned Holstein calves, thus validating its importance in BRD [29].

*NME7*, located on BTA16, was a leading-edge gene in 20 gene sets with a BRD-associated SNP in intron 5. *NME7* causes primary ciliary dyskinesia, a condition that leads to improper cilia formation and function throughout the airways and therefore improper mucus transport [82,83,84]. Regulation and proper formation of airway cilia is essential for correct movement of mucus throughout the lungs, where the failure to do so could lead to chronic pulmonary complications and potentially susceptibility to respiratory disease.

Two genes, *TFEC* and *FHIT*, were identified in three of the four analyses performed within this study, highlighting their significance with BRD in this population. *TFEC*, has been previously identified as a candidate gene for bovine tuberculosis in African zebu cattle [85]. *TFEC* has also been identified as a Hub gene and is differentially expressed for infection of BRSV and IBR among beef cattle [30,34]. Specifically, *TFEC* has been found to activate macrophages and dendritic cells in mice, as *TFEC* increases the expression of IL-4, leading to M2 (alternative) programming in macrophages, where the M2 macrophage phenotype is characterized by anti-inflammatory processes [86,87,88]. *FHIT* has been previously identified as a leading-edge gene for feedlot beef cattle with BRD [29]. *FHIT*’s specific role within BRD is still largely unknown, though it has been found to be highly expressed within circulating monocytes and pulmonary macrophages, suggesting important functional roles directly in immune cells and as a factor of the MHC [89]. The validation of these genes highlights the importance of their role in BRD.

There were 633 genes that were shared between this study and previous BRD studies (Supplemental Table S4). *PLCB1* was identified in post-weaned heifers as a leading-edge gene and in four previous studies as a leading-edge gene in beef cattle, as a differentially expressed gene in BRD infected cattle, and as a positional candidate gene in pre-weaned dairy cattle in Italy [29,30,31,34]. *PLCB1* has also been associated with chronic obstructive pulmonary disease and endothelial cell inflammation [90,91]. *PLCB1* is downregulated in patients with COVID-19 and was an enriched gene for inflammatory processes gene sets and is predicted to activate macrophages and B cells [92]. The repeated identification of *PLCB1* in beef and dairy cattle with different pathogens highlights the potential of this gene to be used for selection for more BRD resistant cattle and warrants further examination into its role within BRD.

## 5. Conclusions

This study identified 25 loci strongly (*P* < 5 × 10^−7^) and 37 loci moderately (*P* < 1 × 10^−5^) associated with BRD in pre-weaned heifers and 89 loci strongly (*P* < 5 × 10^−7^) and 92 loci moderately (*P* < 1 × 10^−5^) associated with BRD in post-weaned heifers. There were 40 unique positional candidate genes associated (*P* < 1 × 10^−5^) with BRD in pre-weaned heifers where the BRD-associated SNP was within an intron or exon, and 67 unique positional candidate genes were similarly identified as associated (*P* < 1 × 10^−5^) with BRD in the post-weaned heifers. There were 12 enriched gene sets (NES ≥ 3.0) with 126 unique leading-edge genes enriched with BRD in the pre-weaned heifers, and 63 gene sets with 849 unique leading-edge genes enriched (NES ≥ 3.0) with BRD in the post-weaned heifers. There were 66 positional candidate genes, 551 leading-edge genes, and 16 genes that were identified as both positional candidate and leading-edge genes within this study, as well as five gene sets that have previously been reported to be associated with or enriched for BRD. Twenty-two leading edge genes identified in this study have been previously identified in three or more BRD studies. These loci and genes should be further investigated to identify how they function in the etiology of this disease and should be considered for predictors for susceptibility to BRD.

## Figures and Tables

**Table 1:** The three most significant loci for additive, dominant and recessive inheritance models and their respective positional candidate genes associated with bovine respiratory disease in pre-weaned Holstein heifers.

Inheritance Model ^1^	*p*-Value ^2^	BTA ^3^	Mb ^4^	No. Associated SNPs ^5^	Positional Candidate Genes ^6^
Additive	6.37 × 10^−13^	29	25	13	*CSRP3*, *E2F8*, *IGSF22*, *LOC507011*, *MRGPRX2*, *PTPN5*, *SPTY2D1*, *SPTY2D1OS*, *TMEM86A*, *UEVLD*, *ZDHHC13*
Additive	1.31 × 10^−11^	2	114	1	-
Additive	7.48 × 10^−11^	10	12	1	***DENND4A***, *LOC104973042*
Dominant	1.09 × 10^−11^	29	26	11	*CSRP3*, *E2F8*, *IGSF22*, *MRGPRX2*, *PTPN5*, *SPTY2D1*, *SPTY2D1OS*, *TMEM86A*, *UEVLD*, *ZDHHC13*
Dominant	2.02 × 10^−10^	10	12	1	***DENND4A***, *LOC104973042*
Dominant	2.80 × 10^−8^	19	66	1	***LOC512869***, *TRNAG-CCC*
Recessive	7.66 × 10^−14^	2	28	1	-
Recessive	1.40 × 10^−10^	14	33–36	2	***WWP1***
Recessive	7.41 × 10^−9^	4	114	6	*MACC1*, *LOC101907567*, ***LOC104968411***, *TRNAC-GCA*

^1^ The inheritance model where each locus was associated with pre-weaned BRD. ^2^ The uncorrected *p*-value for the lead SNP at each locus. ^3^ *Bos taurus* chromosome (BTA) where the locus is associated (*P* < 5 × 10^−7^) with pre-weaned BRD. ^4^ Megabase (Mb) position of each locus. ^5^ Number (No.) of single nucleotide polymorphisms (SNPs) present within each BRD associated locus. ^6^ Positional candidate genes are genes that were identified within ±29 kb (5′ and 3′) of associated SNPs. Bolded positional candidate genes have one of the BRD-associated SNPs located within an intron or exon.

**Table 2:** The three most significant loci for additive, dominant, and recessive inheritance models and the positional candidate genes in those loci associated with bovine respiratory disease in post-weaned Holstein heifers.

Inheritance Model ^1^	*p*-Value ^2^	BTA ^3^	Mb ^4^	No. Associated SNPs ^5^	Positional Candidate Genes ^6^
Additive	3.17 × 10^−17^	26	42	8	*DMBT1*, *FAM24A*, *LOC100295742*, ***LOC100849037***, *LOC510536*, *LOC517971*, ***LOC617705***
Additive	4.60 × 10^−15^	6	114	2	***ABLIM2***, *MIR95*
Additive	1.72 × 10^−14^	29	12	2	***DLG2***
Dominant	6.79 × 10^−19^	26	42	8	*DMBT1*, *FAM24A*, *LOC100295742*, ***LOC100849037***, *LOC510536*, ***LOC617705***
Dominant	1.11 × 10^−14^	29	12	2	***DLG2***
Dominant	7.04 × 10^−13^	20	66	6	*LOC112443052*, *LOC112443058*, *MED10*, *PAPD7*
Recessive	2.39 × 10^−19^	4	28	7	*MACC1*
Recessive	1.02 × 10^−17^	4	33–36	17	***KIAA1324L***, *LOC112446533*, *LOC781303*, ***SEMA3A*, *SEMA3D***
Recessive	2.01 × 10^−17^	6	114	2	***ABLIM2***, *MIR95*

^1^ The inheritance model where each locus was associated with post-weaned BRD. ^2^ The uncorrected *p*-value for the lead SNP at each locus. ^3^ *Bos taurus* chromosome (BTA) number of where the locus is associated with pre-weaned BRD. ^4^ Megabase (Mb) position of each locus. ^5^ Number (No.) of single nucleotide polymorphisms (SNPs) associated (*P* < 1 × 10^−5^) with BRD in each locus. ^6^ Positional candidate genes are genes that were identified within ± 29 kb (5′ and 3′) of BRD associated (*P* < 1 × 10^−5^) SNPs. Bolded positional candidate genes have one SNP associated (*P* < 1 × 10^−5^) with BRD located within an intron or exon.

**Table 3:** The five gene sets with the largest normalized enrichment scores for bovine respiratory disease in the gene set enrichment analysis–single nucleotide polymorphism for pre-weaned and post-weaned Holstein heifers.

Gene Set (Database) ^1^	NES ^2^	No. LEG ^3^	Leading-Edge Genes ^4^
**Pre-Weaned BRD**			
Negative regulation of protein kinase activity (GO)	3.92	19	*APOE*, *DUSP10*, *GADD45G*, *GSTP1*, *HEXIM1*, *HEXIM2*, *IGF1R*, *IL1B*, *ITGB1BP1*, *MEN1*, *NPM1*, *PLK1*, *PRKAR2A*, *PRKRIP1*, *PSEN1*, *SOCS2*, *SOCS5*, *STK38*, *TRIB2*
Cardiac muscle tissue development (GO)	3.84	6	*CSRP3*, *FOXP1*, *MYL3*, *RBPJ*, *SMAD1*, *TGFB2*
Negative regulation of cell cycle (GO)	3.63	22	*BRINP1*, *CDC5L*, *DDX39B*, *E2F8*, *FAP*, *HEXIM1*, *HEXIM2*, *HPGD*, *IL12B*, *LCMT1*, *MEN1*, *MLF1*, *MSH2*, *OVOL1*, *PKD2*, *PLK1*, *RHOB*, *TEX14*, *TGFB1*, *TGFB2*, *ZWILCH*, *ZWINT*
Negative regulation of kinase activity (GO)	3.56	19	*APOE*, *DUSP10*, *GADD45G*, *GSTP1*, *HEXIM1*, *HEXIM2*, *IGF1R*, *IL1B*, *ITGB1BP1*, *MEN1*, *NPM1*, *PLK1*, *PRKAR2A*, *PRKRIP1*, *PSEN1*, *SOCS2*, *SOCS5*, *STK38*, *TRIB2*
DNA replication (GO)	3.54	33	*CDK2*, *DNA2*, *DTD1*, *E2F8*, *FHIT*, *GINS3*, *GINS4*, *IGF1R*, *KITLG*, *MCIDAS*, *MCM3*, *MEN1*, *MMS22L*, *NFIB*, *ORC2*, *ORC3*, *PDGFA*, *POLA2*, *POLD1*, *POLD3*, *POLD4*, *POLE2*, *POLG2*, *PRIMPOL*, *RBBP4*, *RBMS1*, *RFC3*, *RMI1*, *SMARCAL1*, *SMC3*, *TGFB1*, *TIPIN*, *ZPR1*
**Post-Weaned BRD**			
Purine Metabolism (KEGG)	4.43	39	*ADCY2*, *ADCY3*, *ADCY4*, *AK4*, *AK5*, *AK7*, *AMPD2*, *ENTPD1*, *ENTPD5*, *ENTPD6*, *ENTPD8*, *FHIT*, *GMPR*, *GMPR2*, *GUCY2C*, *GUCY2F*, *IMPDH1*, *NME6*, *NME7*, *PDE1C*, *PDE3A*, *PDE4B*, *PDE4D*, *PDE6A*, *PDE9A*, *POLD2*, *POLR1C*, *POLR2B*, *POLR2C*, *POLR2K*, *POLR3A*, *POLR3B*, *POLR3C*, *POLR3G*, *POLR3GL*, *POLR3K*, *PRIM2*, *RRM2*, *ZNRD1*
Cytosolic DNA sensing pathway (KEGG)	4.17	16	*IL1B*, *IL6*, *NFKB1*, *NFKBIB*, *POLR1C*, *POLR3A*, *POLR3B*, *POLR3C*, *POLR3G*, *POLR3GL*, *POLR3K*, *RIPK1*, *RIPK3*, *TBK1*, *TMEM173*, *TREX1*
Organonitrogen compound metabolic process (GO)	3.98	141	*AARSD1*, *AASS*, *ABAT*, *ABCG2*, *ABHD12*, *ABHD3*, *ACPP*, *ADAL*, *ADCY1*, *ADM*, *ADRB3*, *AK4*, *AK5*, *ALAS1*, *ALDH6A1*, *ALDH7A1*, *APOE*, *ASNS*, *ASNSD1*, *ASS1*, *ATP5S*, *ATP6V1A*, *BDH2*, *BLVRB*, *BTBD9*, *CDO1*, *CHIA*, *CHID1*, *CHPT1*, *COL4A3BP*, *COQ9*, *CPQ*, *CREM*, *CROT*, *CTSH*, *CXCL10*, *CXCL11*, *CXCL9*, *DARS2*, *DCXR*, *DDAH2*, *DDC*, *DEGS1*, *DEGS2*, *DHPS*, *DLST*, *DPYD*, *DRD1*, *DRD5*, *ECE1*, *EDNRA*, *ENOPH1*, *ENOSF1*, *ENTPD5*, *EXT2*, *FAH*, *FECH*, *FHIT*, *FXN*, *G6PC*, *GAL3ST1*, *GATA3*, *GATC*, *GBA*, *GGT7*, *GLCE*, *GLUD1*, *GMPR2*, *GNG7*, *GNS*, *GOT1L1*, *GOT2*, *GPLD1*, *GPX1*, *GSTA2*, *GSTA4*, *GUCY2F*, *HAGH*, *HIBCH*, *HPX*, *IL1B*, *IMPDH1*, *IP6K3*, *ITIH1*, *ITIH3*, *ITIH4*, *KCNAB2*, *KDSR*, *LYVE1*, *MGST2*, *MMAB*, *MSH2*, *MTHFD1L*, *MTRR*, *NADSYN1*, *NARS*, *NDST2*, *NDUFS1*, *NME7*, *NOS3*, *NUDT12*, *OAT*, *ODC1*, *OPRM1*, *OVGP1*, *PAM*, *PAOX*, *PEMT*, *PHGDH*, *PKD2*, *PNPO*, *PPARGC1A*, *PSEN1*, *PTDSS1*, *PTGDR*, *PTH*, *SARS*, *SARS2*, *SERINC1*, *SHMT2*, *SIRT4*, *SLC25A25*, *SMPD1*, *SMPDL3A*, *SMS*, *SNCA*, *SOD1*, *SPCS1*, *SPCS3*, *SPTLC1*, *ST6GALNAC6*, *STAT5B*, *TALDO1*, *TBPL1*, *TCN2*, *TGFB1*, *TH*, *TYMS*, *UMPS*, *WARS*, *XDH*
Cell cycle (R)	3.96	109	*ALMS1*, *ANAPC1*, *ANAPC4*, *ATR*, *ATRIP*, *B9D2*, *BRCA1*, *BUB1*, *BUB3*, *CCND2*, *CDC14A*, *CDC25C*, *CDC45*, *CDK4*, *CENPA*, *CENPN*, *CENPO*, *CEP192*, *CHEK2*, *CLASP1*, *CUL1*, *DIDO1*, *DYNC1H1*, *DYRK1A*, *E2F2*, *E2F3*, *GINS1*, *GINS2*, *HAUS2*, *HIST1H2BA*, *HIST1H2BB*, *HIST1H2BI*, *HIST1H2BJ*, *HIST1H2BL*, *HIST1H2BN*, *HIST2H2AA4*, *HIST2H2AC*, *HIST2H2BE*, *KIF18A*, *KIF23*, *LIN52*, *LIN54*, *MAD1L1*, *MCM3*, *MCM7*, *MIS18A*, *MIS18BP1*, *MYC*, *NDEL1*, *NEDD1*, *OFD1*, *ORC3*, *ORC6*, *PCNT*, *POLD2*, *POT1*, *PPP2R5A*, *PPP2R5C*, *PPP2R5D*, *PPP2R5E*, *PRIM2*, *PRKAR2B*, *PSMA5*, *PSMA6*, *PSMB10*, *PSMB5*, *PSMB8*, *PSMB9*, *PSMC3*, *PSMC4*, *PSMC5*, *PSMC6*, *PSMD12*, *PSMD4*, *PSMD7*, *PSMD8*, *PSME1*, *PSME2*, *PSME4*, *PTTG1*, *RANGAP1*, *RB1*, *REC8*, *RFC2*, *RFC3*, *RPA1*, *RPA3*, *RRM2*, *SEH1L*, *SSNA1*, *STAG1*, *STAG2*, *STAG3*, *SYCP3*, *SYNE1*, *SYNE2*, *TERF1*, *TERF2*, *TFDP1*, *TINF2*, *TUBB*, *TUBG1*, *TUBGCP6*, *TYMS*, *UBE2I*, *WEE1*, *XPO1*, *ZW10*, *ZWILCH*
Purine ribonucleoside metabolic process (GO)	3.95	18	*ADAL*, *AK4*, *AK5*, *ATP5S*, *ATP6V1A*, *COQ9*, *CROT*, *ENTPD5*, *FXN*, *GMPR2*, *IMPDH1*, *IP6K3*, *NME7*, *PPARGC1A*, *PTGDR*, *SLC25A25*, *SNCA*, *TGFB1*

^1^ Database sources; GO = Gene Ontology, KEGG = Kyoto Encyclopedia of Genes and Genomes, R = Reactome. ^2^ NES is the normalized enrichment score. ^3^ Number (No.) of leading-edge genes (LEGs) enriched for bovine respiratory disease in each gene set. ^4^ Names of leading-edge genes.

**Table 4:** Bovine respiratory disease positional candidate and/or leading-edge genes shared among genomic analyses and/or between the pre-weaned and post-weaned age groups.

Age and Analysis Where Genes Were Shared ^1^	No. Genes ^2^	Positional Candidate or Leading-Edge Genes ^3^
PCG & LEG in Pre-Weaned Heifers	1	*SOCS5*
PCG & LEG in Post-Weaned Heifers	11	*ALMS1*, *C6*, *ENTPD5*, *FHIT*, *LOC534742*, *NME7*, *SEMA3C*, *STAG1*, *SYNDIG1L*, *TFEC*, *XPO1*
PCG in Pre- and Post-Weaned Heifers	16	*GPNMB*, *IGF2BP3*, *LOC100138586*, *LOC101907567*, *LOC101907914*, ***LOC104968411***, *LOC107132385*, *LOC107132393*, *MACC1*, *MALSU1*, *NUPL2*, ***RAPGEF5***, ***TENM3***, ***TFEC***, *TRNAC-GCA*, *TRNAK-UUU*
LEG in Pre- and Post-Weaned Heifers	36	*APOE*, *ARL3*, *ARRB1*, *C4A*, *CFB*, *CFH*, *CSRP3*, *DCPS*, *DDX39B*, *DUSP10*, *E2F8*, *FHIT*, *FOXP1*, *IL12B*, *IL1B*, *ISG15*, *LTA*, *LTF*, *MCM3*, *MSH2*, *NOD2*, *ORC3*, *PKD2*, *PSEN1*, *RBM22*, *RBM4*, *RBPJ*, *RFC3*, *RHOA*, *SMARCAL1*, *SOCS2*, *TGFB1*, *TGFB2*, *TNF*, *USP4*, *ZWILCH*

^1^ The age of heifers and the analyses where the genes were shared. PCG = positional candidate gene. LEG = leading-edge gene. ^2^ The number (No.) of genes that were shared with the two analyses. ^3^ Positional candidate genes that were shared with the two analyses. Bolded positional candidate genes have a BRD-associated SNP located within it.

## Data Availability

Data generated and/or analyzed during the current study are pending review and acceptance by the USDA Ag Data Commons (https://figshare.com/s/a6978c716b25f1562f1a (accessed on 3 June 2025)).

## Supplementary Materials

The following supporting information can be downloaded at: https://www.mdpi.com/article/10.3390/genes16070741/s1, Supplemental Table S1. Results for the genome wide association analysis of pre-weaned Holstein heifer calves listing loci associated (*P* < 1 × 10^−5^) with bovine respiratory disease. Supplemental Table S2. Results for the genome wide association analysis of post-weaned Holstein heifer calves listing loci associated (*P* < 1 × 10^−5^) with bovine respiratory disease. Supplemental Table S3. Results of gene set enrichment analysis—single nucleotide polymorphism analysis for pre-weaned and post-weaned Holstein heifer calves. Supplemental Table S4. Genes associated with bovine respiratory disease that are shared in the current and previous bovine respiratory disease studies. Supplemental Table S5. Gene sets enriched with bovine respiratory disease that are shared in the current and previous bovine respiratory disease studies.
