# Supplementary material for: Genomic Regions Associated with Respiratory Disease in Holstein Calves in the Southern United States
Source: Genes (Basel). 2025 Jun 26;16(7):741. doi: 10.3390/genes16070741 (PMC12294756; doi:10.3390/genes16070741)
Supplement: Supplementary file 1 [file genes-16-00741-s001.zip › genes-3710353-supplementary.pdf]

Supplemental Table S1. Results for the genome wide association analysis of pre-weaned Holstein heifer calves listing loci associated ($P < 1 \times 10^{-5}$) with bovine respiratory disease.

BTA[1]	# Associated SNPs[2]	Mb[3]	P-Value[4]	Inheritance Model[5]	Positional Candidate Genes[6]
1	1	39	2.68×10^{-6}	Additive	-
1	1	61	8.09×10^{-6}	Additive	***LSAMP***, *TRNAK-UUU*
1	1	82	8.90×10^{-6}	Additive	***C1H3orf70***, *LOC112447474, TRNAK-UUU, VPS8*
2	1	113	2.33×10^{-6}	Additive	*DOCK10*
2	1	114	**1.31×10^{-11}**	Additive	-
3	2	57	**1.46×10^{-8}**	Additive	***CLCA3***, *CLCA4*, ***LOC112446001***, *LOC784768, TRNAC-GCA*
3	6	121	**2.13×10^{-7}**	Additive	***2-Sep***, ***FARP2***, *HDLBP*
4	2	52	1.21×10^{-6}	Additive	***TFEC***
7	4	97	**4.42×10^{-8}**	Additive	-
7	1	108	5.18E-06	Additive	***FER***
7	2	108	5.68×10^{-6}	Additive	***FER***
8	2	22	5.90×10^{-7}	Additive	*LOC782525*
9	3	39	**4.65×10^{-7}**	Additive	*FYN, GTF3C6, LOC112448037, LOC112448038, LOC112448182, LOC512978, LOC616830,* ***LOC784866,*** *MIR6536-1, MIR6536-2, REV3L,* ***RPF2, TRAF3IP2***
10	1	13	**7.48×10^{-11}**	Additive	***DENND4A***, *LOC104973042*
11	1	33	7.54×10^{-6}	Additive	***NRXN1***
11	1	34	3.51×10^{-6}	Additive	-

11	1	78	6.99×10^{-6}	Additive	*APOB*
11	1	96	$\mathbf{1.51 \times 10^{-7}}$	Additive	*LOC100848407, LOC107133462, LOC112448901, NR6A1, OLFML2A*
12	1	67	3.26×10^{-6}	Additive	***GPC5***
14	3	74	6.60×10^{-7}	Additive	*LOC100848958, LOC101906456,* ***LOC112449547,*** *NBN, OSGIN2, RIPK2*
14	2	76	$\mathbf{1.27 \times 10^{-8}}$	Additive	***WWP1***
19	1	52	3.77×10^{-6}	Additive	***LOC512869****, TRNAG-CCC*
22	1	17	$\mathbf{2.79 \times 10^{-7}}$	Additive	***IRAK2****, LOC101906342, LOC112443428, TATDN2*
27	1	2	9.36×10^{-6}	Additive	***CSMD1****,* ***LOC112444582***
29	13	26	$\mathbf{6.37 \times 10^{-13}}$	Additive	*CSRP3, E2F8, IGSF22, LOC507011, MRGPRX2, PTPN5, SPTY2D1, SPTY2D1OS, TMEM86A, UEVLD, ZDHHC13*
X	4	54	2.32×10^{-6}	Additive	***IL1RAPL2****, LOC784210*
1	1	39	$\mathbf{1.16 \times 10^{-7}}$	Dominant	-
1	3	40	4.55×10^{-6}	Dominant	-
1	1	61	9.41×10^{-6}	Dominant	***LSAMP****, TRNAK-UUU*
1	1	131	8.13×10^{-6}	Dominant	-
3	2	57	$\mathbf{1.19 \times 10^{-7}}$	Dominant	***CLCA3,*** *CLCA4****,*** ***LOC112446001,*** *LOC784768, TRNAC-GCA*
3	6	121	1.43×10^{-6}	Dominant	***2-Sep****,* ***FARP2****, HDLBP*
7	3	97	3.82×10^{-6}	Dominant	-
7	1	108	4.49×10^{-6}	Dominant	***FER***
8	1	22	9.80×10^{-7}	Dominant	*LOC782525*

9	3	39	1.16×10^{-6}	Dominant	*FYN, GTF3C6, LOC112448037, LOC112448038, LOC112448182, LOC512978, LOC616830,* ***LOC784866,*** *MIR6536-1, MIR6536-2, REV3L,* ***RPF2, TRAF3IP2***
10	1	13	$\mathbf{2.02 \times 10^{-10}}$	Dominant	***DENND4A****, LOC104973042*
11	1	29	1.57×10^{-6}	Dominant	*LOC112448772,* ***SOCS5***
11	1	96	$\mathbf{1.78 \times 10^{-7}}$	Dominant	*LOC100848407, LOC107133462, LOC112448901, NR6A1, OLFML2A*
12	1	67	$\mathbf{3.41 \times 10^{-7}}$	Dominant	***GPC5***
14	1	65	7.89×10^{-7}	Dominant	*MIR599, MIR875,* ***VPS13B***
14	5	74	$\mathbf{4.35 \times 10^{-7}}$	Dominant	*LOC100848958, LOC101906456,* ***LOC112449547,*** *LOC112449548, NBN, OSGIN2,* ***RIPK2***
16	1	15	6.81×10^{-6}	Dominant	***BRINP3***
19	1	52	$\mathbf{2.80 \times 10^{-8}}$	Dominant	***LOC512869,*** *TRNAG-CCC*
22	1	17	$\mathbf{4.69 \times 10^{-7}}$	Dominant	***IRAK2,*** *LOC101906342, LOC112443428, TATDN2*
27	4	3	1.88×10^{-6}	Dominant	***CSMD1, LOC112444582***
27	1	14	3.89×10^{-6}	Dominant	***TENM3,*** *TRNAC-ACA*
27	3	18	6.62×10^{-6}	Dominant	*LOC507011*
29	11	26	$\mathbf{1.09 \times 10^{-11}}$	Dominant	*CSRP3, E2F8, IGSF22, MRGPRX2, PTPN5, SPTY2D1, SPTY2D1OS, TMEM86A, UEVLD, ZDHHC13*
2	1	114	$\mathbf{7.66 \times 10^{-14}}$	Recessive	**-**
4	6	30	$\mathbf{7.41 \times 10^{-9}}$	Recessive	*LOC101907567,* ***LOC104968411****, MACC1, TRNAC-GCA*

3	4	31	**3.19×10^{-7}**	Recessive	*GPNMB, IGF2BP3, LOC100138586, LOC101907914, LOC101907978, LOC107132385, LOC107132393, LOC112446363, MALSU1, NUPL2,* ***RAPGEF5***
4	5	39	5.96×10^{-6}	Recessive	*LOC107132393*
4	12	94	4.52×10^{-6}	Recessive	*IRF5, CHCHD3,* ***COPG2, EXOC4,*** *KCP, LOC112446418, LOC112446420,* ***MARK4,*** *MEST, TNPO3, TRNAS-GGA*
6	1	117	3.83×10^{-6}	Recessive	*LETM1, LOC101905114,* ***NSD2***
7	4	97	**5.51×10^{-8}**	Recessive	-
12	3	63	**3.42×10^{-7}**	Recessive	-
14	2	76	**1.40×10^{-10}**	Recessive	***WWP1***
15	14	52	8.07×10^{-6}	Recessive	*ANAPC15,* ***CLPB, FOLR1, FOLR2,*** *FOLR3, IL18BP,* ***INPPL1,*** *LAMTOR1,* ***LOC112441638,*** *LRRC51, NUMA1,* ***PHOX2A, RNF121,*** *TOMT, TRNAG-CCC*
20	2	6	1.04×10^{-6}	Recessive	*C20H5orf47, LOC104975196, LOC782443,* ***NSG2***
21	3	3	1.30×10^{-6}	Recessive	-
29	1	26	2.54×10^{-6}	Recessive	*CSRP3, E2F8, ZDHHC13*

[1]*Bos taurus* (BTA) chromosome of the locus associated with bovine respiratory disease (BRD) in pre-weaned heifers. [2]Number of single nucleotide polymorphisms (SNPs) present within each BRD associated locus. [3]Megabase (Mb) position of each locus on the chromosome. [4]The uncorrected p-value for the lead SNP for each locus. Bolded values were strongly associated ($P < 5 \times 10^{-7}$) with BRD in pre-weaned heifers. [5]The inheritance model for the association with BRD. [6]Positional candidate genes were identified within ± 29 kb (5′ and 3′) of a BRD-associated SNP. Bolded positional candidate genes have one of the associated SNPs located within the intron or exon of the gene.

Supplemental Table S2. Results for the genome wide association analysis of post-weaned Holstein heifer calves listing loci associated ($P < 1 \times 10^{-5}$) with bovine respiratory disease.

BTA[1]	# Associated SNPs[2]	Mb[3]	P-Value[4]	Inheritance Model[5]	Positional Candidate Genes[6]
1	4	39	5.88×10^{-7}	Additive	-
1	2	45	**1.98×10^{-7}**	Additive	***ABI3BP***
1	1	73	2.78×10^{-6}	Additive	*LOC112447381, LSG1, TMEM44*
1	3	108	4.01×10^{-6}	Additive	***SCHIP1***
1	1	118	3.46×10^{-6}	Additive	-
1	5	132	9.93×10^{-7}	Additive	*LOC112448286,* ***STAG1***
2	7	53	6.69×10^{-6}	Additive	***ARHGAP15***
2	5	84	1.86×10^{-6}	Additive	***DNAH7***
3	1	21	4.38×10^{-6}	Additive	***ANKRD35****, ITGA10, LOC789231, PEX11B, PIAS3*
3	2	35	1.12×10^{-6}	Additive	***LOC534742***
3	1	40	**2.45×10^{-7}**	Additive	-
3	1	89	6.27×10^{-7}	Additive	***DAB1***
4	8	24	**4.86×10^{-8}**	Additive	***AGMO***
4	1	29	5.38×10^{-7}	Additive	*MACC1*
4	10	33-37	**2.81×10^{-9}**	Additive	***KIAA1324L,*** *LOC101904927, LOC112446533, LOC781303,* ***PCLO, SEMA3A, SEMA3D***
4	5	38	3.95×10^{-6}	Additive	*PCLO*
4	17	53	**5.00×10^{-9}**	Additive	***TFEC***
4	1	89	**1.76×10^{-7}**	Additive	*LOC112446338*

4	5	90	5.77×10^{-6}	Additive	-
4	2	90	**3.04×10^{-9}**	Additive	-
5	2	49	2.00×10^{-6}	Additive	***RASSF3***
5	1	64	1.22×10^{-6}	Additive	-
5	1	77	2.20×10^{-6}	Additive	-
6	2	5	7.56×10^{-7}	Additive	*PRDM5*
6	1	99	6.51×10^{-6}	Additive	-
6	2	99	**2.65×10^{-12}**	Additive	-
6	2	114	**4.60×10^{-15}**	Additive	***ABLIM2***, *MIR95*
6	2	115	**3.25×10^{-8}**	Additive	***SH3TC1***
6	7	115	**7.80×10^{-10}**	Additive	*ACOX3*, ***HTRA3***
7	1	51	8.42×10^{-6}	Additive	*UBE2D2*
8	1	5	6.27×10^{-6}	Additive	***GALNTL6***
8	2	9	**3.33×10^{-10}**	Additive	***KIF13B***
8	1	45	**2.78×10^{-7}**	Additive	***TJP2***
9	1	54	7.04×10^{-6}	Additive	***MANEA***
9	2	67	**1.55×10^{-7}**	Additive	*LOC104969587*, ***PTPRK***
10	1	42	**3.03×10^{-7}**	Additive	-
10	5	58	**5.51×10^{-8}**	Additive	*FAM214A*
10	1	85	7.85×10^{-7}	Additive	*BBOF1*, *COQ6*, ***ENTPD5***
10	1	86	**4.45×10^{-7}**	Additive	***SYNDIG1L***

10	1	97	1.43×10^{-6}	Additive	-
11	2	11	9.82×10^{-6}	Additive	***ALMS1,*** *NAT8*
11	1	11	8.25×10^{-6}	Additive	*ALMS1*
11	3	49-51	$\mathbf{3.91 \times 10^{-7}}$	Additive	*ATOH8*
11	7	60	$\mathbf{1.74 \times 10^{-7}}$	Additive	***LOC112448801, USP34, XPO1***
11	1	73	1.14×10^{-6}	Additive	*ADGRF3, HADHB, LOC101906131*
12	1	40	5.07×10^{-7}	Additive	***PCDH9***
13	1	25	3.13×10^{-6}	Additive	***KIAA1217***, *LOC107133036, LOC112449393*
14	6	61	$\mathbf{2.21 \times 10^{-8}}$	Additive	***BAALC,*** *CTHRC1,* ***FZD6,*** *LOC783954*
14	1	77	8.73×10^{-6}		-
15	3	48-49	$\mathbf{2.95 \times 10^{-8}}$	Additive	*LOC100847455, LOC100847801, LOC511823, LOC528807, LOC530994, LOC531174, LOC614922, LOC615276, LOC617817, LOC785207, LOC785238, LOC787971, LOC787988, LOC788024, OR52D1*
15	1	67	4.90×10^{-6}	Additive	-
15	20	67	$\mathbf{2.46 \times 10^{-9}}$	Additive	-
15	2	68	6.31×10^{-7}	Additive	-
15	1	80	$\mathbf{3.32 \times 10^{-11}}$	Additive	*LOC614143*
16	1	37	$\mathbf{1.28 \times 10^{-9}}$	Additive	***NME7***
16	1	46	$\mathbf{4.17 \times 10^{-9}}$	Additive	***CAMTA1***
16	11	52	$\mathbf{3.39 \times 10^{-11}}$	Additive	***FHAD1, TMEM51***
17	2	37	6.17×10^{-6}	Additive	-

18	1	4	**1.69 x 10^{-8}**	Additive	-
18	1	4	**7.60 x 10^{-11}**	Additive	-
18	8	49	**1.35 x 10^{-7}**	Additive	*ACP7, LOC112442313*
18	1	53	2.18 x 10^{-6}	Additive	*BCAM, CBLC, LOC100141014, LOC104974889, NECTIN2*
19	3	38	1.29 x 10^{-6}	Additive	*CBX1, CDK5RAP3,* ***LOC112442655,*** *NFE2L1, PNPO, PRR15L,* ***SKAP1,*** *SNX11, SP2*
20	3	33	**1.66 x 10^{-7}**	Additive	***C6***
20	6	67	**1.54 x 10^{-12}**	Additive	*LOC112443052, LOC112443058, MED10, PAPD7*
21	1	22	1.83 x 10^{-6}	Additive	***CRTC3****, TRNAW-CCA*
21	1	25	2.60 x 10^{-6}	Additive	*BNC1*
21	2	40	**4.69 x 10^{-10}**	Additive	***PRKD1***
22	1	40	7.36 x 10^{-6}	Additive	***FHIT***
22	7	41	**5.19 x 10^{-9}**	Additive	***FHIT***
23	2	10	**8.66 x 10^{-8}**	Additive	***FKBP5***
23	2	31	**6.88 x 10^{-8}**	Additive	*MIR7857,* ***PRSS16,*** *TRNAD-GUC, TRNAI-AAU, TRNAI-UAU, TRNAK-UUU, TRNAL-CAA, TRNAL-UAA, TRNAM-CAU, TRNAQ-CUG, TRNAR-ACG, TRNAR-UCU, TRNAS-AGA, TRNAS-CGA, TRNAS-UGA, TRNAT-AGU, TRNAT-CGU, TRNAV-CAC, TRNAV-UAC*
26	9	12-26	**3.00 x 10^{-7}**	Additive	**-**
26	8	42	**3.17 x 10^{-17}**	Additive	*DMBT1, FAM24A, LOC100295742,* ***LOC100849037,*** *LOC510536,* ***LOC617705***
27	4	5	**3.54 x 10^{-11}**	Additive	*LOC517971*

27	14	5	6.68×10^{-7}	Additive	-
27	1	13	3.02×10^{-6}	Additive	***TENM3***
29	3	1	$\mathbf{8.60 \times 10^{-9}}$	Additive	-
29	2	12	$\mathbf{1.72 \times 10^{-14}}$	Additive	***DLG2***
29	1	12	1.24×10^{-6}	Additive	*DLG2*
29	1	26	$\mathbf{4.21 \times 10^{-7}}$	Additive	-
X	3	40	$\mathbf{6.08 \times 10^{-9}}$	Additive	*PCDH11X*
X	3	115	2.12×10^{-6}	Additive	-
X	1	134	8.79×10^{-6}	Additive	-
1	3	40	1.78×10^{-6}	Dominant	-
1	2	45	$\mathbf{6.02 \times 10^{-8}}$	Dominant	***ABI3BP***
1	2	108	5.41×10^{-6}	Dominant	***SCHIP1***
1	2	132	1.53×10^{-6}	Dominant	*LOC112448286*
1	3	132-147	1.03×10^{-6}	Dominant	***STAG1***
2	1	18	6.53×10^{-6}	Dominant	*LOC112443667*, ***OSBPL6***
3	2	35-36	$\mathbf{2.15 \times 10^{-7}}$	Dominant	***LOC534742***
3	1	40	$\mathbf{3.56 \times 10^{-7}}$	Dominant	-
4	2	34	$\mathbf{6.36 \times 10^{-8}}$	Dominant	*LOC101904927*
4	17	53	$\mathbf{2.01 \times 10^{-7}}$	Dominant	***TFEC***
4	3	80	$\mathbf{5.12 \times 10^{-8}}$	Dominant	*LOC112446338*
5	1	72	6.54×10^{-6}	Dominant	*LARGE1*

6	2	5	5.76×10^{-6}	Dominant	*PRDM5*
6	1	99	**5.49×10^{-9}**	Dominant	-
6	1	114	**5.00×10^{-7}**	Dominant	***ABLIM2***, *MIR95*
6	2	115	**1.38×10^{-7}**	Dominant	***SH3TC1***
7	1	51	4.33×10^{-6}	Dominant	*UBE2D2*
8	1	9	**7.70×10^{-8}**	Dominant	***KIF13B***
8	2	45	**5.34×10^{-8}**	Dominant	***TJP2***
10	6	58	**2.80×10^{-7}**	Dominant	*FAM214A*
10	1	85	5.82×10^{-7}	Dominant	*BBOF1, COQ6,* ***ENTPD5***
10	1	86	1.20×10^{-6}	Dominant	***SYNDIG1L***
10	1	97	2.82×10^{-6}	Dominant	**-**
11	2	9	3.77×10^{-6}	Dominant	*LOC101909815, POU3F3*
11	2	11	1.05×10^{-6}	Dominant	***ALMS1,*** *NAT8*
11	1	11	4.67×10^{-6}	Dominant	***ALMS1***
11	2	52	2.97×10^{-6}	Dominant	**-**
11	3	60	**3.39×10^{-7}**	Dominant	***LOC112448801***
11	1	73	3.82×10^{-6}	Dominant	*ADGRF3, HADHB, LOC101906131*
12	1	40	**2.72×10^{-7}**	Dominant	***PCDH9***
13	1	25	**1.19×10^{-7}**	Dominant	***KIAA1217,*** *LOC107133036, LOC112449393*
14	3	61	**2.14×10^{-10}**	Dominant	***BAALC, FZD6,*** *LOC783954*
14	1	77	4.12×10^{-6}	Dominant	**-**

15	1	48	4.98×10^{-6}	Dominant	*LOC100847801, LOC531174, LOC614922, LOC787971, LOC787988, LOC788024*
15	1	67	3.28×10^{-6}	Dominant	**-**
15	16	67	**4.82×10^{-8}**	Dominant	**-**
15	1	68	4.82×10^{-6}	Dominant	-
15	1	80	**3.91×10^{-11}**	Dominant	*LOC614143*
16	1	37	**6.95×10^{-11}**	Dominant	***NME7***
16	1	46	**4.16×10^{-9}**	Dominant	***CAMTA1***
16	11	52	**3.17×10^{-11}**	Dominant	***FHAD1, TMEM51***
17	3	37	1.53×10^{-6}	Dominant	**-**
18	1	4	**5.82×10^{-8}**	Dominant	**-**
18	1	4	**4.90×10^{-8}**	Dominant	**-**
18	8	48	7.71×10^{-7}	Dominant	*ACP7, LOC112442313*
18	1	53	1.36×10^{-6}	Dominant	*BCAM, CBLC, LOC100141014, LOC104974889, NECTIN2*
19	2	38	1.18×10^{-6}	Dominant	*CDK5RAP3,* ***LOC112442655,*** *PNPO, PRR15L,* ***SKAP1,*** *SP2*
20	1	33	1.80×10^{-6}	Dominant	***C6***
20	1	44	3.79×10^{-6}	Dominant	*LOC521159*
20	6	66	**7.04×10^{-13}**	Dominant	*LOC112443052, LOC112443058, MED10, PAPD7*
21	6	24-44	**2.51×10^{-8}**	Dominant	*BNC1, LOC784193,* ***NPAS3, PRKD1***
21	4	54	2.12×10^{-6}	Dominant	***LOC112443191***
22	5	41	3.44×10^{-6}	Dominant	***FHIT***
23	2	10	**6.18×10^{-9}**	Dominant	***FKBP5***

23	1	31	**1.63×10^{-7}**	Dominant	***PRSS16,*** *TRNAI-AAU, TRNAI-AAU, TRNAI-AAU, TRNAI-AAU, TRNAL-UAA, TRNAM-CAU, TRNAR-ACG, TRNAR-ACG, TRNAS-CGA, TRNAT-AGU, TRNAT-AGU, TRNAV-CAC, TRNAV-CAC, TRNAV-UAC*
23	8	36	4.02×10^{-6}	Dominant	***LOC104968751***
24	1	12	**2.27×10^{-8}**	Dominant	-
26	3	26	2.48×10^{-6}	Dominant	-
26	8	42	**4.02×10^{-13}**	Dominant	*DMBT1, FAM24A, LOC100295742,* ***LOC100849037,*** *LOC510536,* ***LOC617705***
27	3	5	**5.71×10^{-11}**	Dominant	-
27	1	13	1.80×10^{-6}	Dominant	***TENM3***
29	2	1	**3.89×10^{-9}**	Dominant	-
29	2	12	**1.11×10^{-14}**	Dominant	***DLG2***
29	1	12	7.25×10^{-7}	Dominant	*DLG2*
29	1	25	1.75×10^{-6}	Dominant	-
X	3	40	**8.94×10^{-8}**	Dominant	*PCDH11X*
X	3	115	1.61×10^{-6}	Dominant	-
2	1	114	6.48×10^{-6}	Recessive	-
3	1	89	2.36×10^{-6}	Recessive	*C8A*
4	23	20-24	**6.79×10^{-9}**	Recessive	***AGMO, DGKB, THSD7A,*** *TRNAC-GCA*
4	1	26	3.27×10^{-7}	Recessive	*LOC101902492, SNX13*
4	7	28	**2.39×10^{-19}**	Recessive	*MACC1*

4	1	28	**1.59×10^{-8}**	Recessive	*MACC1*
4	2	30	6.12×10^{-7}	Recessive	***LOC104971984,*** *LOC613345*
4	14	30	**2.21×10^{-16}**	Recessive	***CDCA7L***, *LOC101907567*, ***LOC104968411***
4	5	31	**1.32×10^{-13}**	Recessive	*LOC100138586*, *LOC101907914*, ***LOC107132385, RAPGEF5***
4	2	31	**8.00×10^{-10}**	Recessive	***GPNMB,*** *IGF2BP3, MALSU1, NUPL2*
4	4	32	7.40×10^{-7}	Recessive	***IGF2BP3,*** *TRA2A*
4	1	33	1.45×10^{-6}	Recessive	*CROT*
4	17	33-36	**1.02×10^{-17}**	Recessive	***KIAA1324L,*** *LOC112446533*, *LOC781303*, ***SEMA3D***
4	25	37-40	**1.35×10^{-13}**	Recessive	*LOC107132393*, ***PCLO, SEMA3C***
4	1	94	2.17×10^{-6}	Recessive	*MIR182, MIR183, MIR96*, ***NRF1***
5	1	64	3.10×10^{-6}	Recessive	**-**
6	2	114	**2.01×10^{-17}**	Recessive	***ABLIM2,*** *MIR95*
6	7	114	**5.03×10^{-12}**	Recessive	*ACOX3*, ***HTRA3***
8	3	9	**3.67×10^{-7}**	Recessive	***KIF13B***
8	1	85	4.19×10^{-6}	Recessive	***LOC787410***
9	2	66	1.88×10^{-6}	Recessive	*LOC104969587*, ***PTPRK***
11	1	43	2.22×10^{-6}	Recessive	**-**
15	1	48	2.39×10^{-6}	Recessive	*LOC528807, LOC530994, LOC615276, LOC617817, OR52D1*
17	5	33	**1.99×10^{-7}**	Recessive	**-**
18	1	4	3.08×10^{-6}	Recessive	**-**
20	1	33	**5.36×10^{-8}**	Recessive	***C6***

20	1	66	5.88×10^{-6}	Recessive	*PAPD7*
22	2	41	1.67×10^{-6}	Recessive	***FHIT***
23	2	31	3.73×10^{-6}	Recessive	***ABT1,*** *LOC101902221, LOC514235, LOC521167, TRNAA-AGC, TRNAN-GUU, TRNAY-GUA, TRNAY-GUA, TRNAY-GUA*
28	5	16	5.15×10^{-7}	Recessive	***RHOBTB1***

[1]*Bos taurus* (BTA) chromosome of the locus associated with BRD in post-weaned heifers. [2]Number of single nucleotide polymorphisms (SNPs) present within each BRD associated locus. [3]Megabase (Mb) position of each locus on the chromosome. [4]The uncorrected p-value for the lead SNP for each locus. Bolded values were strongly associated ($P < 5 \times 10^{-7}$) with BRD in post-weaned heifers. [5]The inheritance model for the association with BRD. [6]Positional candidate genes were identified within ± 29 kb (5′ and 3′) of a BRD associated SNP. Bolded positional candidate genes have one of the associated SNPs located within the intron or exon of the gene.

Supplemental Table S3. Results of gene set enrichment analysis – single nucleotide polymorphism analysis for pre-weaned and post-weaned Holstein heifer calves.

Enriched Gene Sets	Database[1]	NES[2]	Functional Group[3]	# LEG[4]	Leading Edge Genes[5]
**Pre-Weaned BRD**					
Negative regulation of protein kinase activity	GO	3.92	1	19	***APOE***, *DUSP10*, *GADD45G*, *GSTP1*, *HEXIM1*, *HEXIM2*, *IGF1R*, ***IL1B***, *ITGB1BP1*, <u>*MEN1*</u>, *NPM1*, <u>*PLK1*</u>, *PRKAR2A*, *PRKRIP1*, *PSEN1*, ***SOCS2***, *SOCS5*, *STK38*, *TRIB2*
Cardiac muscle tissue development	GO	3.84	2	6	***CSRP3***, ***FOXP1***, *MYL3*, ***RBPJ***, *SMAD1*, ***TGFB2***
Negative regulation of cell cycle	GO	3.63	1	22	*BRINP1*, *CDC5L*, ***DDX39B***, ***E2F8***, *FAP*, *HEXIM1*, *HEXIM2*, *HPGD*, ***IL12B***, *LCMT1*, <u>*MEN1*</u>, *MLF1*, ***MSH2***, *OVOL1*, ***PKD2***, <u>*PLK1*</u>, *RHOB*, *TEX14*, ***TGFB1***, ***TGFB2***, ***ZWILCH***, *ZWINT*
Negative regulation of kinase activity	GO	3.56	1	19	***APOE***, ***DUSP10***, *GADD45G*, *GSTP1*, *HEXIM1*, *HEXIM2*, *IGF1R*, ***IL1B***, *ITGB1BP1*, <u>*MEN1*</u>, *NPM1*, <u>*PLK1*</u>, *PRKAR2A*, *PRKRIP1*, ***PSEN1***, ***SOCS2***, *SOCS5*, *STK38*, *TRIB2*
DNA replication	GO	3.54	3	33	*CDK2*, *DNA2*, *DTD1*, ***E2F8***, ***FHIT***, *GINS3*, *GINS4*, *IGF1R*, *KITLG*, *MCIDAS*, ***MCM3***, <u>*MEN1*</u>, *MMS22L*, *NFIB*, *ORC2*, ***ORC3***, *PDGFA*, *POLA2*, *POLD1*, *POLD3*, *POLD4*, *POLE2*, *POLG2*, *PRIMPOL*, *RBBP4*, *RBMS1*, ***RFC3***, *RMI1*, ***SMARCAL1***, *SMC3*, ***TGFB1***, *TIPIN*, *ZPR1*
Cytokinesis	GO	3.51	2	10	***ARL3***, *CFL1*, ***E2F8***, *NUSAP1*, <u>*PLK1*</u>, *RASA1*, ***RHOA***, *RHOB*, *SPAST*, *TEX14*
Negative regulation of transferase activity	GO	3.49	1	19	***APOE***, *DUSP10*, *GADD45G*, *GSTP1*, *HEXIM1*, *HEXIM2*, *IGF1R*, ***IL1B***, *ITGB1BP1*, <u>*MEN1*</u>, *NPM1*, <u>*PLK1*</u>, *PRKAR2A*, *PRKRIP1*, ***PSEN1***, ***SOCS2***, *SOCS5*, *STK38*, *TRIB2*
Cardiocyte differentiation	GO	3.46	2	4	***CSRP3***, *FOXP1*, ***RBPJ***, ***TGFB2***

RNA splicing via transesterification reactions & mRNA splicing via spliceosome & RNA splicing via transesterification reactions with bulged adenosine as nucleophile	GO	3.33	3	31	*CDC5L*, *CDK13*, ***DCPS***, ***DDX39B***, *GEMIN6*, *GEMIN7*, *GEMIN8*, *HNRNPA2B1*, *LSM8*, *MAGOH*, *NCBP2*, *PQBP1*, *PRMT5*, *PRMT7*, *PRPF4*, ***RBM22***, ***RBM4***, *RBM8A*, *RBMXL2*, *RSRC1*, *SF3B3*, *SLU7*, *SMN2*, *SNRNP70*, *SNRPC*, *SNRPE*, *SRSF6*, *SRSF7*, *STRAP*, *TFIP11*, ***USP4***
Negative regulation of protein modification process	GO	3.31	1	28	***APOE***, ***ARRB1***, *ATG5*, *DNMT1*, *DUSP10*, *GADD45G*, *GSTP1*, *HEXIM1*, *HEXIM2*, *IGF1R*, ***IL1B***, ***ISG15***, *ITGB1BP1*, <u>*MEN1*</u>, *NPM1*, <u>*PLK1*</u>, *PRKAR2A*, *PRKRIP1*, ***PSEN1***, ***SOCS2***, *SOCS5*, *SPINK1*, *STK38*, ***TGFB1***, *TRIB2*, *TRIM21*, *TRIP12*, ***USP4***
Humoral immune response	GO	3.16	4	14	*BMI1*, *C2*, ***C4A***, *C4BPA*, *C4BPB*, *CD46*, ***CFB***, ***CFH***, ***LTA***, ***LTF***, *MBL2*, ***NOD2***, ***RBPJ***, ***TNF***
Cardiac muscle cell differentiation	GO	3.16	2	3	***CSRP3***, ***FOXP1***, ***RBPJ***
**Post-Weaned BRD**					
Purine Metabolism	KEGG	4.43	5	39	*ADCY2*, *ADCY3*, *ADCY4*, *AK4*, *AK5*, *AK7*, *AMPD2*, *ENTPD1*, *ENTPD5*, *ENTPD6*, *ENTPD8*, ***FHIT***, *GMPR*, *GMPR2*, *GUCY2C*, *GUCY2F*, *IMPDH1*, *NME6*, *NME7*, *PDE1C*, *PDE3A*, *PDE4B*, *PDE4D*, *PDE6A*, *PDE9A*, *POLD2*, *POLR1C*, *POLR2B*, *POLR2C*, *POLR2K*, *POLR3A*, *POLR3B*, *POLR3C*, *POLR3G*, *POLR3GL*, *POLR3K*, *PRIM2*, *RRM2*, *ZNRD1*
Cytosolic DNA sensing pathway	KEGG	4.17	6	16	***IL1B***, *IL6*, *NFKB1*, *NFKBIB*, *POLR1C*, *POLR3A*, *POLR3B*, *POLR3C*, *POLR3G*, *POLR3GL*, *POLR3K*, *RIPK1*, *RIPK3*, *TBK1*, *TMEM173*, *TREX1*
Organonitrogen compound metabolic process	GO	3.98	5	141	*AARSD1*, *AASS*, *ABAT*, *ABCG2*, *ABHD12*, *ABHD3*, *ACPP*, *ADAL*, *ADCY1*, *ADM*, *ADRB3*, *AK4*, *AK5*, *ALAS1*, *ALDH6A1*, *ALDH7A1*, ***APOE***, *ASNS*, *ASNSD1*, *ASS1*, *ATP5S*, *ATP6V1A*, *BDH2*, *BLVRB*, *BTBD9*, *CDO1*, *CHIA*, *CHID1*, *CHPT1*, *COL4A3BP*, *COQ9*, *CPQ*, *CREM*, *CROT*, *CTSH*,

					CXCL10, CXCL11, CXCL9, DARS2, DCXR, DDAH2, DDC, DEGS1, DEGS2, DHPS, DLST, DPYD, DRD1, DRD5, ECE1, EDNRA, ENOPH1, ENOSF1, ENTPD5, EXT2, FAH, FECH, ***FHIT****, FXN, G6PC, GAL3ST1, GATA3, GATC, GBA, GGT7, GLCE, GLUD1, GMPR2, GNG7, GNS, GOT1L1, GOT2, GPLD1, GPX1, GSTA2, GSTA4, GUCY2F, HAGH, HIBCH, HPX,* ***IL1B****, IMPDH1, IP6K3, ITIH1, ITIH3, ITIH4, KCNAB2, KDSR, LYVE1, MGST2, MMAB, MSH2, MTHFD1L, MTRR, NADSYN1, NARS, NDST2, NDUFS1, NME7, NOS3, NUDT12, OAT, ODC1, OPRM1, OVGP1, PAM, PAOX, PEMT, PHGDH,* ***PKD2****, PNPO, PPARGC1A,* ***PSEN1****, PTDSS1, PTGDR, PTH, SARS, SARS2, SERINC1, SHMT2, SIRT4, SLC25A25, SMPD1, SMPDL3A, SMS, SNCA, SOD1, SPCS1, SPCS3, SPTLC1, ST6GALNAC6, STAT5B, TALDO1, TBPL1, TCN2,* ***TGFB1****, TH, TYMS, UMPS, WARS, XDH*
Cell cycle	R	3.96	3	109	*ALMS1, ANAPC1, ANAPC4, ATR, ATRIP, B9D2, BRCA1, BUB1, BUB3, CCND2, CDC14A, CDC25C, CDC45, CDK4, CENPA, CENPN, CENPO, CEP192, CHEK2, CLASP1, CUL1, DIDO1, DYNC1H1, DYRK1A, E2F2, E2F3, GINS1, GINS2, HAUS2, HIST1H2BA, HIST1H2BB, HIST1H2BI, HIST1H2BJ, HIST1H2BL, HIST1H2BN, HIST2H2AA4, HIST2H2AC, HIST2H2BE, KIF18A, KIF23, LIN52, LIN54, MAD1L1,* ***MCM3****, MCM7, MIS18A, MIS18BP1, MYC, NDEL1, NEDD1, OFD1,* ***ORC3****, ORC6, PCNT, POLD2, POT1, PPP2R5A, PPP2R5C, PPP2R5D, PPP2R5E, PRIM2, PRKAR2B, PSMA5, PSMA6, PSMB10, PSMB5, PSMB8, PSMB9, PSMC3, PSMC4, PSMC5, PSMC6, PSMD12, PSMD4, PSMD7, PSMD8, PSME1, PSME2, PSME4, PTTG1, RANGAP1, RB1, REC8, RFC2,* ***RFC3****, RPA1, RPA3, RRM2, SEH1L, SSNA1, STAG1, STAG2, STAG3, SYCP3, SYNE1, SYNE2, TERF1, TERF2, TFDP1, TINF2, TUBB, TUBG1, TUBGCP6, TYMS, UBE2I, WEE1, XPO1, ZW10,* ***ZWILCH***

Purine ribonucleoside metabolic process	GO	3.95	5	18	*ADAL, AK4, AK5, ATP5S, ATP6V1A, COQ9, CROT, ENTPD5, FXN, GMPR2, IMPDH1, IP6K3, NME7, PPARGC1A, PTGDR, SLC25A25,* <u>*SNCA*</u>, <u>***TGFB1***</u>
Nucleoside monophosphate metabolic process	GO	3.92	5	18	*AK4, AK5, ATP5S, ATP6V1A, COQ9, DPYD, ENTPD5, FXN, GMPR2, IMPDH1, IP6K3,* ***MSH2****, PPARGC1A, SLC25A25,* <u>*SNCA*</u>, <u>***TGFB1***</u>, *TYMS, UMPS*
Chromosome maintenance	R	3.91	3	29	*CENPA, CENPO, DIDO1, HIST1H2BA, HIST1H2BB, HIST1H2BJ, HIST1H2BL, HIST1H2BN, HIST2H2AA4, HIST2H2AC, HIST2H2BE, MIS18A, MIS18BP1, POLD2, POT1, PRIM2, REC8,* ***RFC3****, RPA1, RPA3, STAG1, STAG2, STAG3, SYNE1, SYNE2, TERF1, TERF2, TINF2, UBE2I*
Purine nucleoside monophosphate metabolic process & purine ribonucleoside monophosphate metabolic process	GO	3.89	5	15	*AK4, AK5, ATP5S, ATP6V1A, COQ9, ENTPD5, FXN, GMPR2, IMPDH1, IP6K3, MSH2, PPARGC1A, SLC25A25,* <u>*SNCA*</u>, <u>***TGFB1***</u>
Purine ribonucleoside triphosphate metabolic process	GO	3.79	5	14	*AK4, AK5, ATP5S, ATP6V1A, COQ9, ENTPD5, FXN, IP6K3,* ***MSH2****, NME7, PPARGC1A, SLC25A25,* <u>*SNCA*</u>, <u>***TGFB1***</u>
Purine nucleoside metabolic process	GO	3.77	5	18	*ADAL, AK4, AK5, ATP5S, ATP6V1A, COQ9, CROT, ENTPD5, FXN, GMPR2, IMPDH1, IP6K3, NME7, PPARGC1A, PTGDR, SLC25A25,* <u>*SNCA*</u>, <u>***TGFB1***</u>
Purine nucleoside triphosphate metabolic process	GO	3.76	5	14	*AK4, AK5, ATP5S, ATP6V1A, COQ9, ENTPD5, FXN, IP6K3,* ***MSH2****, NME7, PPARGC1A, SLC25A25,* <u>*SNCA*</u>, <u>***TGFB1***</u>
Ribonucleoside triphosphate metabolic process	GO	3.74	5	14	*AK4, AK5, ATP5S, ATP6V1A, COQ9, ENTPD5, FXN, IP6K3,* ***MSH2****, NME7, PPARGC1A, SLC25A25,* <u>*SNCA*</u>, <u>***TGFB1***</u>
Response to bacterium	GO	3.72	7	61	*AKIRIN2, APOA2, B2M, CAMP, CATHL1, CATHL3, CATHL4, CHGA, CXCL10, CXCL11, CXCL16, CXCL2, CXCL8, CXCL9, DEFB1, DEFB10,*

					DEFB4A, DEFB7, ***DUSP10****, FCER1G, H2B, IFNG, IL10,* ***IL12B****,* ***IL1B****, IL6, IRAK2,* ***ISG15****, LAP, LIAS, LPO,* ***LTA****,* ***LTF****, LYZ1, LYZ2, LYZ3, MAPK1, MAPKAPK3, MEF2C, MR1, NCF1, NLRP3,* ***NOD2****, NR1H3, PENK, PLAC8,* ***RBPJ****, ROMO1, SEH1L, SERPINE1, SNCA,* ***TGFB1****, TICAM2, TLR2, TLR4, TLR6,* ***TNF****, TNFAIP8, TRAF6, XBP1, ZC3H12A*
Purine nucleotide metabolic process	GO	3.72	5	35	*ADCY1, ADM, ADRB3, AK4, AK5,* ***APOE****, ATP5S, ATP6V1A, COQ9, CROT, CXCL10, CXCL11, CXCL9, DRD1, DRD5, EDNRA, ENTPD5,* ***FHIT****, FXN, GMPR2, GNG7, GUCY2F, IMPDH1, IP6K3,* ***MSH2****, NDUFS1, NME7, NOS3, OPRM1,* ***PKD2****, PPARGC1A, PTH, SLC25A25, SNCA,* ***TGFB1***
Ribonucleoside monophosphate metabolic process	GO	3.70	5	17	*AK4, AK5, ATP5S, ATP6V1A, COQ9, DPYD, ENTPD5, FXN, GMPR2, IMPDH1, IP6K3,* ***MSH2****, PPARGC1A, SLC25A25, SNCA,* ***TGFB1****, UMPS*
Meiosis	R	3.67	3	35	*ATR, BRCA1, CDK4, DIDO1, HIST1H2BA, HIST1H2BB, HIST1H2BI, HIST1H2BJ, HIST1H2BL, HIST1H2BN, HIST1H3C, HIST1H3G, HIST1H3I, HIST2H2AA4, HIST2H2AC, HIST2H2BE, MLH1, MSH5, NBN, POT1, RAD51, RBBP8, REC8, RPA1, RPA3, STAG1, STAG2, STAG3, SYCP3, SYNE1, SYNE2, TERF1, TERF2, TINF2, UBE2I*
Ribonucleoside metabolic process	GO	3.66	5	22	*ACPP, ADAL, AK4, AK5, ATP5S, ATP6V1A, COQ9, CROT, DPYD, ENTPD5, FXN, GMPR2, IMPDH1, IP6K3,* ***MSH2****, NME7, PPARGC1A, PTGDR, SLC25A25, SNCA,* ***TGFB1****, UMPS*
Nucleoside triphosphate metabolic process	GO	3.64	5	16	*AK4, AK5, ATP5S, ATP6V1A, COQ9, ENTPD5, FXN, IP6K3,* ***MSH2****, NME7, PPARGC1A, SLC25A25, SNCA, TBPL1,* ***TGFB1****, TYMS*
Purine containing compound metabolic process	GO	3.63	5	46	*ABCG2, ACPP, ADAL, ADCY1, ADM, ADRB3, AHCY, AK4, AK5, APIP,* ***APOE****, ATP5S, ATP6V1A, COQ9, CROT, CTNS, CXCL10, CXCL11, CXCL9, DRD1, DRD5, EDNRA, ENTPD5,* ***FHIT****, FXN, G6PC, GMPR2, GNG7, GUCY2F, IMPDH1, IP6K3, MCEE,* ***MSH2****, NDUFS1, NME7,*

					NOS3, OAS2, OPRM1, ***PKD2****, PPARGC1A, PTGDR, PTH, SLC25A25,* <u>*SNCA*</u>*,* <u>***TGFB1***</u>*, XDH*
Response to biotic stimulus	GO	3.61	7	79	*AKIRIN2, AP1S1, APOA2, B2M, CAMP, CATHL1, CATHL3, CATHL4, CHGA, CXCL10, CXCL11, CXCL16, CXCL2, CXCL8, CXCL9, DDIT3, DDX1, DEFB1, DEFB10, DEFB4A, DEFB7,* ***DUSP10****, ELMOD2, FCER1G, GATA3, H2B, IFNAR1, IFNG, IFNT3, IL10,* ***IL12B****, IL15,* ***IL1B****, IL6, IRAK2, IRF5,* ***ISG15****, LAP, LIAS, LPO,* ***LTA****,* ***LTF****, LYZ1, LYZ2, LYZ3, MAPK1, MAPKAPK3, MEF2C, MR1, NCF1,* ***NOD2****, NPC2, OAS2, PENK, PLAC8, POLR3A, POLR3C, POLR3K,* ***RBPJ****, ROMO1, SEH1L, SERPINE1,* <u>*SNCA*</u>*, SYNDIG1L,* <u>***TGFB1***</u>*, TICAM1, TICAM2, TLR2, TLR4, TLR6, TMEM173,* ***TNF****, TNFAIP8, TRAF6, TRIM11, TRIM38, TRIM56, XBP1, ZC3H12A*
Pyrophosphatase activity	GO	3.60	8	77	*ABCC1, ABCF2, ABCG2, ALPL, ARL2,* ***ARL3****, ASB14, ASCC3, ATL1, ATP1A1, ATP1B3, ATP6V1A, ATP6V1E2, ATP6V1F, ATP6V1H, ATP8A1, ATP8A2, CFTR, CHD1L,* ***DCPS****, DDX1, DDX25,* ***DDX39B****, DNAI1, DNAJC27, DNM1L, DXO, DYNC1I1, DYNC2LI1, DYNLRB1, DYNLRB2, EEF1A2, EEF2, EFTUD2, ENTPD5, ERCC6L2,* ***FHIT****, GNAO1, GNAT2, GNG2, GPN1, GTF2F2, GTF2H2, HBS1L, KIF3C, KLC4, LHPP, LONP2,* ***MCM3****, MCM7, MOV10,* ***MSH2****, MTG1, MYL6, MYO1A, MYO1D, NUDT12, NUDT3, PPA1, RAB11A, RAB27B, RAB30, RAB3C, RAB5C, RAB6B, RAC1, RAD51, RAN, RAP1A, RFC2,* ***RFC3****, RHOU, SMARCAD1,* ***SMARCAL1****, TUBB, TUBB2B, TUBG1*
Hydrolase activity acting on acid anhydrides in phosphorus containing anhydrides	GO	3.60	8	78	*ABCC1, ABCF2, ABCG2, ACYP2, ALPL, ARL2,* ***ARL3****, ASB14, ASCC3, ATL1, ATP1A1, ATP1B3, ATP6V1A, ATP6V1E2, ATP6V1F, ATP6V1H, ATP8A1, ATP8A2, CFTR, CHD1L,* ***DCPS****, DDX1, DDX25,* ***DDX39B****, DNAI1, DNAJC27, DNM1L, DXO, DYNC1I1, DYNC2LI1, DYNLRB1, DYNLRB2, EEF1A2, EEF2, EFTUD2, ENTPD5, ERCC6L2,* ***FHIT****, GNAO1, GNAT2, GNG2, GPN1, GTF2F2, GTF2H2, HBS1L, KIF3C, KLC4, LHPP, LONP2,* ***MCM3****, MCM7, MOV10,* ***MSH2****, MTG1, MYL6, MYO1A,*

					MYO1D, NUDT12, NUDT3, PPA1, RAB11A, RAB27B, RAB30, RAB3C, RAB5C, RAB6B, RAC1, RAD51, RAN, RAP1A, RFC2, ***RFC3****, RHOU, SMARCAD1,* ***SMARCAL1****, TUBB, TUBB2B, TUBG1*
Small molecule metabolic process	GO	3.59	5	162	*AARSD1, AASS, ABAT, ABCG2, ABHD10, ABHD3, ACADS, ACADSB, ACPP, ADAL, ADCY1, ADM, ADRB3, AFP, AK4, AK5, ALDH6A1, ALDH7A1, ALDH8A1, APOC2,* ***APOE****, ASNS, ASNSD1, ASS1, ATP5S, ATP6V1A, BRCA1, CA1, CA3, CAT, CBR1, CDO1, CHPT1, COQ2, COQ4, COQ6, COQ9, CPT1B, CROT, CS, CXCL10, CXCL11, CXCL9, CYB5R1, CYP11A1, CYP17A1, DARS2, DCXR, DDAH2, DDC, DEGS1, DEGS2, DGAT2, DHCR7, DLST, DPYD, DRD1, DRD5, EDNRA, ELOVL5, ENOPH1, ENOSF1, ENTPD5, ETFDH, EXT2, FAH, FAM213B, FASN, FDFT1, FDX1, FECH,* ***FHIT****, FXN, G6PC, GADL1, GAPDH, GAPDHS, GATA3, GATC, GLCE, GLUD1, GMPR2, GNG7, GNPAT, GOT1L1, GOT2, GPLD1, GUCY2F, HADHB, HAO2, HIBCH, HK1, HMGCL, HMGCS2, HTR2A, IFNG,* ***IL1B****, IMPDH1, IP6K3, ITPK1, KCNAB2, LOC782922, LPO, MBTPS2, MGST2, MMAB, MPC1,* ***MSH2****, MTHFD1L, MTRR, MVD, MVK, NADSYN1, NARS, NDST2, NDUFS1, NME7, NOS3, NUDT12, NUDT3, OAT, ODC1, OPRM1, PEMT, PHGDH,* ***PKD2****, PLIN5, PLP1, PNPO, PPARGC1A, PTGDR, PTGES, PTGES2, PTGS1, PTH, RDH10, SARS, SARS2, SCAP, SCD5, SHMT2, SIRT4, SLC25A25, SMPD1, SMPDL3A, <u>SNCA</u>, SOD1, SPTLC1, STAT5B, SUCLA2, TALDO1, TBPL1, TCN2,* ***<u>TGFB1</u>****, TH, THEM4, TM7SF2, TXN2, TYMS, UMPS, WARS, XDH*
Defense response	GO	3.58	7	124	*A2M, ADORA1, ADORA3, AIF1, AKIRIN2, APOA2,* ***APOE****, ATG12, BDNF,* ***C4A****, C6, CAMP, CATHL1, CATHL3, CATHL4, CCL11, CCRL2, CEBPG,* ***CFB****,* ***CFH****, CHGA, CHIA, CHID1, CNPY3, COLEC12, CTSS, CXCL10, CXCL11, CXCL16, CXCL2, CXCL8, CXCL9, CYBA, CYBB, DEFB1, DEFB10, DEFB4A, DEFB7, DRD1,* ***DUSP10****, ELMOD2, F3, FCER1G, FEM1A, FN1, FYN, GATA3, GHRL, GNG7, GRO1, H2B, HPX, IFNAR1, IFNAR2, IFNG, IFNT3, IL10,* ***IL12B****, IL15,* ***IL1B****, IL1RN, IL34,*

					IL6, IRAK2, IRF5, ***ISG15****, ITGB6, ITIH4, LAMP1, LAP, LGR4, LIAS, LPO,* ***LTA****,* ***LTF****, LYST, LYZ1, LYZ2, LYZ3, MAPKAPK3, MEF2C, MR1, NCF1, NFKBIZ, NLRP3, NOD2, NR1H3, NRROS, OAS2, PENK, PLAC8, PLP1, POLR3A, POLR3C, POLR3K, PTGDR, PTGIS, PTGS1, PYCARD, RABGEF1,* ***RBPJ****, RIPK2, ROMO1, SAA2, SAA4, SEH1L, SERPINE1,* <u>*SNCA*</u>*, STAT5B,* <u>***TGFB1***</u>*, TICAM1, TICAM2, TLR10, TLR2, TLR4, TLR6, TMEM173,* ***TNF****, TNFAIP8, TNFAIP8L2, TOLLIP, TRIM11, TRIM38, TRIM56*
Hydrolase activity acting on acid anhydrides	GO	3.54	8	78	*ABCC1, ABCF2, ABCG2, ACYP2, ALPL, ARL2,* ***ARL3****, ASB14, ASCC3, ATL1, ATP1A1, ATP1B3, ATP6V1A, ATP6V1E2, ATP6V1F, ATP6V1H, ATP8A1, ATP8A2, CFTR, CHD1L,* ***DCPS****, DDX1, DDX25,* ***DDX39B****, DNAI1, DNAJC27, DNM1L, DXO, DYNC1I1, DYNC2LI1, DYNLRB1, DYNLRB2, EEF1A2, EEF2, EFTUD2, ENTPD5, ERCC6L2,* ***FHIT****, GNAO1, GNAT2, GNG2, GPN1, GTF2F2, GTF2H2, HBS1L, KIF3C, KLC4, LHPP, LONP2,* ***MCM3****, MCM7, MOV10,* ***MSH2****, MTG1, MYL6, MYO1A, MYO1D, NUDT12, NUDT3, PPA1, RAB11A, RAB27B, RAB30, RAB3C, RAB5C, RAB6B, RAC1, RAD51, RAN, RAP1A, RFC2, RFC3, RHOU, SMARCAD1,* ***SMARCAL1****, TUBB, TUBB2B, TUBG1*
Pyrimidine metabolism	KEGG	3.51	5	28	*CAD, DPYD, ENTPD1, ENTPD5, ENTPD6, ENTPD8, NME3, NME6, NME7, POLD2, POLR1C, POLR2B, POLR2C, POLR2F, POLR2K, POLR3A, POLR3B, POLR3C, POLR3G, POLR3GL, POLR3K, PRIM2, RRM2, TYMS, UCKL1, UMPS, UPB1, ZNRD1*
Ribonucleotide metabolic process	GO	3.49	5	36	*ADCY1, ADM, ADRB3, AK4, AK5,* ***APOE****, ATP5S, ATP6V1A, COQ9, CROT, CXCL10, CXCL11, CXCL9, DPYD, DRD1, DRD5, EDNRA, ENTPD5, FXN, GMPR2, GNG7, GUCY2F, IMPDH1, IP6K3,* ***MSH2****, NDUFS1, NME7, NOS3, OPRM1,* ***PKD2****, PPARGC1A, PTH, SLC25A25,* <u>*SNCA*</u>*,* <u>***TGFB1***</u>*, UMPS*

Glycosyl compound metabolic process	GO	3.49	5	21	*ABHD10, ADAL, AK4, AK5, ATP5S, ATP6V1A, COQ9, CROT, DPYD, ENTPD5, FUCA1, FXN, GMPR2, IMPDH1, IP6K3, NME7, PPARGC1A, PTGDR, SLC25A25, <u>SNCA</u>,* ***<u>TGFB1</u>***
Meiotic synapsis	R	3.49	3	20	*DIDO1, HIST1H2BA, HIST1H2BB, HIST1H2BJ, HIST1H2BL, HIST1H2BN, HIST2H2AA4, HIST2H2AC, HIST2H2BE, POT1, REC8, STAG1, STAG2, STAG3, SYNE1, SYNE2, TERF1, TERF2, TINF2, UBE2I*
Response to other organism	GO	3.48	7	79	*AKIRIN2, AP1S1, APOA2, B2M, CAMP, CATHL1, CATHL3, CATHL4, CHGA, CXCL10, CXCL11, CXCL16, CXCL2, CXCL8, CXCL9, DDX1, DEFB1, DEFB10, DEFB4A, DEFB7,* ***DUSP10****, ELMOD2, FCER1G, GATA3, H2B, IFNAR1, IFNG, IFNT3, IL10,* ***IL12B****, IL15,* ***IL1B****, IL6, IRAK2, IRF5,* ***ISG15****, LAP, LIAS, LPO,* ***LTA****,* ***LTF****, LYZ1, LYZ2, LYZ3, MAPK1, MAPKAPK3, MEF2C, MR1, NCF1, NLRP3,* ***NOD2****, NPC2, NR1H3, OAS2, PENK, PLAC8, POLR3A, POLR3C, POLR3K,* ***RBPJ****, ROMO1, SEH1L, SERPINE1, <u>SNCA</u>,* ***<u>TGFB1</u>****, TICAM1, TICAM2, TLR2, TLR4, TLR6, TMEM173,* ***TNF****, TNFAIP8, TRAF6, TRIM11, TRIM38, TRIM56, XBP1, ZC3H12A*
Purine ribonucleotide metabolic process	GO	3.47	5	34	*ADCY1, ADM, ADRB3, AK4, AK5,* ***APOE****, ATP5S, ATP6V1A, COQ9, CROT, CXCL10, CXCL11, CXCL9, DRD1, DRD5, EDNRA, ENTPD5, FXN, GMPR2, GNG7, GUCY2F, IMPDH1, IP6K3,* ***MSH2****, NDUFS1, NME7, NOS3, OPRM1,* ***PKD2****, PPARGC1A, PTH, SLC25A25, <u>SNCA</u>,* ***<u>TGFB1</u>***
Response to external biotic stimulus	GO	3.39	7	79	*AKIRIN2, AP1S1, APOA2, B2M, CAMP, CATHL1, CATHL3, CATHL4, CHGA, CXCL10, CXCL11, CXCL16, CXCL2, CXCL8, CXCL9, DDX1, DEFB1, DEFB10, DEFB4A, DEFB7,* ***DUSP10****, ELMOD2, FCER1G, GATA3, H2B, IFNAR1, IFNG, IFNT3, IL10,* ***IL12B****, IL15,* ***IL1B****, IL6, IRAK2, IRF5,* ***ISG15****, LAP, LIAS, LPO,* ***LTA****,* ***LTF****, LYZ1, LYZ2, LYZ3, MAPK1, MAPKAPK3, MEF2C, MR1, NCF1, NLRP3,* ***NOD2****, NPC2, NR1H3, OAS2, PENK, PLAC8, POLR3A, POLR3C, POLR3K,* ***RBPJ****, ROMO1, SEH1L,*

					SERPINE1, <u>*SNCA*</u>, <u>***TGFB1***</u>, *TICAM1*, *TICAM2*, *TLR2*, *TLR4*, *TLR6*, *TMEM173*, ***TNF***, *TNFAIP8*, *TRAF6*, *TRIM11*, *TRIM38*, *TRIM56*, *XBP1*, *ZC3H12A*
Hydrolase activity acting on glycosyl bonds	GO	3.38	8	17	*ABHD10*, *ADPRH*, *CHIA*, *CHID1*, *CTBS*, *FUCA1*, *GLB1*, *LYZ1*, *LYZ2*, *LYZ3*, *MAN2B1*, *MANBA*, *OVGP1*, *PARG*, *RPS3*, *SMPD1*, *SMPDL3A*
Proteasome	KEGG	3.37	9	18	*IFNG*, *PSMA5*, *PSMA6*, *PSMB10*, *PSMB11*, *PSMB5*, *PSMB8*, *PSMB9*, *PSMC3*, *PSMC5*, *PSMC6*, *PSMD12*, *PSMD4*, *PSMD7*, *PSMD8*, *PSME1*, *PSME2*, *PSME4*
Cellular response to oxygen containing compound	GO	3.35	7	64	***ARRB1***, *CAPN2*, *CFTR*, *CISH*, *CXCL10*, *CXCL16*, *CXCL8*, ***DCPS***, *DGAT2*, *DMTN*, *DRD1*, *DRD5*, *EIF4EBP1*, *ESR1*, *FXN*, *FYN*, *GH1*, *GNG2*, *GNRHR*, *GPLD1*, *GPR68*, *HSP90B1*, *IFNG*, *IGF2*, ***IL12B***, ***IL1B***, *IL6*, *INHBB*, *IRAK2*, *KANK2*, *LAMTOR4*, *LAMTOR5*, ***LTF***, *MAPK1*, *MEF2C*, *MPV17*, *MYOD1*, *NDOR1*, *NLRP3*, ***NOD2***, *NR1H3*, *OSR1*, *OXR1*, *PIK3R1*, ***PKD2***, *PRKCB*, *PTGDR*, *RAD51*, *RAP1A*, *RAPGEF2*, *RIPK2*, *ROMO1*, *SERPINE1*, ***SOCS2***, *SOD1*, *STAT5B*, <u>***TGFB1***</u>, *TICAM2*, *TLR4*, ***TNF***, *TRAF6*, *TRIB3*, *XBP1*, *ZC3H12A*
ATP metabolic process	GO	3.31	5	3	*ATP5S*, *PPARGC1A*, <u>***TGFB1***</u>
Cytoplasmic vesicle	GO	3.29	10	88	*AP1S1*, *AP2A2*, *AP3B1*, ***ARRB1***, *ATP1B3*, *ATP6V0A1*, *ATP6V1E2*, *ATP8A1*, *BDNF*, *CARTPT*, *CCT4*, *CHGA*, *CHMP4A*, *CHP1*, *CLIC5*, *CLK3*, *CLU*, *COMMD1*, *COPG2*, *COPS4*, *CTSB*, *CTSH*, *CYB561*, *DMTN*, *DNM1L*, *DTNBP1*, *FASN*, *FLOT1*, *GCHFR*, *GLIPR1L1*, *HAX1*, *HCRT*, *HSP90B1*, *HTR2A*, ***IL1B***, *ITPR2*, *ITPR3*, ***LTF***, *MAP1LC3A*, *NAP1L1*, *OVGP1*, *PACSIN1*, *PAM*, *PCSK1*, *PDIA3*, *PIFO*, *PLA1A*, *POMC*, *PPIB*, ***PSEN1***, *PSMC5*, *RAB3C*, *RAB5C*, *RAB7A*, *RAB8B*, *SAYSD1*, *SCAP*, *SCGN*, *SEC23A*, *SERPINA5*, *SLC1A4*, *SLC6A17*, <u>*SNCA*</u>, *SND1*, *SNX17*, *SNX24*, *SOD1*, *SPACA4*, *SPERT*, *SPG21*, *SPX*, *STX19*, *STX1A*, *SV2A*, *SVOP*, *SYT1*, *TBC1D7*, *TLR6*, *TM9SF1*, *TMEM198*, *TMEM30A*, *TRIM9*, *USO1*, *VOPP1*, *VPS51*, *YIPF5*, *YKT6*, *YWHAZ*

DNA replication	R	3.27	3	55	*B9D2, BUB1, BUB3, CDC45, CENPA, CENPN, CENPO, CLASP1, E2F2, E2F3, GINS1, GINS2, KIF18A, KIF23, MAD1L1,* ***MCM3****, MCM7, NDEL1,* ***ORC3****, ORC6, POLD2, PPP2R5A, PPP2R5C, PPP2R5D, PPP2R5E, PRIM2, PSMA5, PSMA6, PSMB10, PSMB5, PSMB8, PSMB9, PSMC3, PSMC4, PSMC5, PSMC6, PSMD12, PSMD4, PSMD7, PSMD8, PSME1, PSME2, PSME4, RANGAP1, RB1, RFC2,* ***RFC3****, RPA1, RPA3, SEH1L, STAG1, STAG2, XPO1, ZW10,* ***ZWILCH***
Cellular response to organonitrogen compound	GO	3.21	7	24	*CAPN2, CISH, DMTN, DRD5, GABRB2, GNG2, GNRHR, GPLD1, HRH1, HSP90B1, IGF2,* ***IL1B****, LAMTOR4, LAMTOR5, MEF2C, OPRM1, PIK3R1, PRKCB, RAD51, RAP1A, RAPGEF2,* ***SOCS2****, STAT5B, XBP1*
Regulation of transport	GO	3.20	1	139	*AAK1, ACTN4, ADORA1, ADORA3, ANG, APOA2,* ***APOE****,* ***ARRB1****, ASPH, ATP1A1, ATP1B3, BTBD9, CABP1, CACNB2, CACNG3, CAPN3, CARTPT, CAV1, CDH3, CDK5, CHGA, CHIA, CHP1, CLCN7, CLIC1, CLIC4, CLIC5, CREBL2, CRYAB, CSN2, CXCL10, CXCL11, CXCL9, CYBA, CYBB, CYLD, DMTN, DNAJC27, DNM1L, DPH3, DPP6, DRD1, DRD2, DTNBP1, EHD1, ENSA, EPO, FCER1G, GAPVD1, GATA3, GH1, GHRL, GLRA1, GLUD1, GOLPH3L, GPLD1, GPR68, HOMER1, HTR2A, IFNAR1, IFNG, IL10,* ***IL1B****, IL6, INHBB, INS, IP6K3, KCNAB1, KCNAB2, KCNB2, KCNH1, KCNIP3, KCNIP4, KCNJ3, KCNK1, KCNMA1, KCNQ3, KCNV1, LAMP1, LGR4, MAPT, MEF2C, NLRP3,* ***NOD2****, NR1H3, NTF3, OPRM1, PACSIN1, PBLD, PDZK1, PFN2, PIK3R1,* ***PKD2****, PLA2R1, PLCB1, POMC, PPID, PRKCB, PRNP, PTH, PYCARD, RAB15, RAB27B, RAB3C, RAB5C, RABGEF1, RAP1A,* ***RBM22****,* ***RBM4****, REEP2, RHBDF1, SAR1A, SCIN, SERGEF, SERPINE1, SIRT4, SLC25A5, SLC8A1, SNCA, SNTA1, SPX, STX1A, SYT1,* ***TGFB1****,* ***TGFB2****, THOC5, TLR10, TLR2, TLR4, TLR6, TMEM173, TMEM30A,* ***TNF****, TNNC1, TRAF6, TRIB3, TRPC6, XBP1, YIPF5*

Response to external stimulus	GO	3.18	7	173	*A2M, ABHD12, ACKR4, ADORA1, ADORA3, ADRB3, AGTR1, AIF1, AKIRIN2, ALB, ALPL, AP1S1, APOA2,* ***APOE*****,** ***ARRB1****, ASNS, ATG101, ATG12, ATG3, B2M, BDNF, CAMP, CAPN3, CARTPT, CATHL1, CATHL3, CATHL4, CAV1, CCL11, CCRL2, CDK5, CHGA, CHMP4A, CMKLR1, CNGA3,* ***CSRP3****, CTSS, CX3CR1, CXCL10, CXCL11, CXCL16, CXCL2, CXCL8, CXCL9, DAP, DDIT3, DDX1, DEFB1, DEFB10, DEFB4A, DEFB7, DMTN,* ***DUSP10****, ECSCR, EDNRB, EFNA1, ELMOD2, ENPP2, ETV1, F12, F7, FABP7, FCER1G, FECH, FEM1A,* ***FOXP1****, FYN, GATA3, GH1, GHRL, GLRA1, GNAT2, GNGT1, GRO1, H2B, HRH1, IFNAR1, IFNG, IFNT3, IL10,* ***IL12B****, IL15,* ***IL1B****, IL6, INHBB, INS, IRAK2, IRF5,* ***ISG15****, ISPD, KANK2, LAP, LEP, LIAS, LPO,* ***LTA*****,** ***LTF****, LYZ1, LYZ2, LYZ3, MAP1LC3A, MAPK1, MAPKAPK3, MEF2C, MR1, MYOD1, NCF1, NENF, NLRP3,* ***NOD2****, NPC2, NPY, NR1H3, NR4A1, NTF3, OAS2, OMA1, OPN1SW, PAFAH1B2, PENK,* ***PKD2****, PLAC8, POLR3A, POLR3C, POLR3K, POMC, PPP1CB, PRKG1, PROS1,* ***PSEN1****, PTGIS, RABGEF1, RAC1,* ***RBM4*****,** ***RBPJ****, RGS9BP,* ***RHOA****, ROMO1, S1PR1, SAA2, SAA4, SCARA5, SEH1L, SEMA3C, SERPINC1, SERPINE1, SLC16A1, SLC1A3, SLC25A25, SNCA, SPX, STAT5B, STRBP, TBC1D14,* ***TGFB1*****,** ***TGFB2****, THBS4, TICAM1, TICAM2, TLR10, TLR2, TLR4, TLR6, TMEM173,* ***TNF****, TNFAIP8, TNFAIP8L2, TRAF6, TRIM11, TRIM38, TRIM56, XBP1, ZC3H12A*
Nucleoside metabolic process	GO	3.17	5	22	*ACPP, ADAL, AK4, AK5, ATP5S, ATP6V1A, COQ9, CROT, DPYD, ENTPD5, FXN, GMPR2, IMPDH1, IP6K3,* ***MSH2****, NME7, PPARGC1A, PTGDR, SLC25A25, SNCA,* ***TGFB1****, UMPS*
Ribose phosphate metabolic process	GO	3.16	5	37	*ADCY1, ADM, ADRB3, AK4, AK5,* ***APOE****, ATP5S, ATP6V1A, COQ9, CROT, CXCL10, CXCL11, CXCL9, DPYD, DRD1, DRD5, EDNRA, ENTPD5, FXN, GMPR2, GNG7, GUCY2F, IMPDH1, IP6K3,* ***MSH2****,*

					NDUFS1, NME7, NOS3, OPRM1, ***PKD2****, PPARGC1A, PTH, SLC25A25,* <u>*SNCA*</u>*, TALDO1,* <u>***TGFB1***</u>*, UMPS*
Regulation of hormone levels	GO	3.16	1	40	*ADORA3, AFP, ALDH8A1,* ***ARRB1****, CARTPT, CHGA, CPQ, CRYM, CYP11A1, CYP17A1, DGAT2, DRD2, ECE1, ENSA, FDX1, GATA3, GH1, GHRL, GLUD1, GPLD1, GPR68, HTR2A, IFNG,* ***IL1B****, IL1RN, IL6, INHBB, INS, IP6K3, NR5A1, POMC, RDH10, RDH8, SIRT4, SRD5A1, STAT5B, SULT1B1, SULT1E1, TG,* ***TNF***
Cellular response to oxidative stress	GO	3.13	7	9	*FXN, IL6, LOC534742, MPV17, NFE2L1, OXR1, PPARGC1A,* <u>*SNCA*</u>*, SOD1*
Mitotic M-M/G1 phases	R	3.13	3	49	*B9D2, BUB1, BUB3, CDC45, CENPA, CENPN, CENPO, CLASP1, E2F2, E2F3, KIF18A, KIF23, MAD1L1,* ***MCM3****, MCM7, NDEL1,* ***ORC3****, ORC6, PPP2R5A, PPP2R5C, PPP2R5D, PPP2R5E, PRIM2, PSMA5, PSMA6, PSMB10, PSMB5, PSMB8, PSMB9, PSMC3, PSMC4, PSMC5, PSMC6, PSMD12, PSMD4, PSMD7, PSMD8, PSME1, PSME2, PSME4, RANGAP1, RPA1, RPA3, SEH1L, STAG1, STAG2, XPO1, ZW10,* ***ZWILCH***
Cysteine type peptidase activity	GO	3.11	8	24	*BAP1, CAPN2, CAPN3, CAPNS1, CASP6, CTSB, CTSC, CTSH, CTSK, CTSS, CYLD, PIGK, RCE1, SENP7, TINAG, UCHL3, UCHL5, USP1, USP10, USP13, USP15, USP20,* ***USP4****, ZRANB1*
Lipid biosynthetic process	GO	3.11	11	74	*AGPAT1, AGPAT4, AJUBA, ALDH8A1, ANG, APOA2, APOC2,* ***APOE****, BRCA1, CD81, CHPT1, COQ2, CREBL2, CYB5R1, CYP11A1, CYP11B1, CYP17A1, CYP21, DEGS1, DEGS2, DGAT2, DHCR7, DPM3, ELOVL5, FAM213B, FASN, FDFT1, FDX1, FITM1, GNPAT, GPLD1, HMGCS2, HSD17B12, HTR2A, IFNG,* ***IL1B****, ISPD, LEP, LIAS, LOC782922, LSS, LTC4S, MGST2, MOGAT1, MVD, MVK, ORMDL1, PEMT, PIGK, PIGY, PIK3CA, PLIN5, PLP1, PTDSS1, PTGES, PTGES2, PTGIS, PTGS1, RDH10, RDH8, SCD5, SDR42E1, SERAC1, SERINC1, SIRT4, SMPD1,*

					SOD1, SPTLC1, SRD5A1, ST6GALNAC6, TAMM41, TM7SF2, ***TNF****, TRIB3*
De novo purine biosynthesis	Panther	3.10	11	10	*AK4, AK8, IMPDH1, KCNC1, KCNC4, NME6, NME7, RPS24, RRM2, TARBP1*
Signaling by WNT	R	3.08	12	24	*APC, CSNK1A1, CUL1, PPP2R5A, PPP2R5C, PPP2R5D, PPP2R5E, PSMA5, PSMA6, PSMB10, PSMB5, PSMB8, PSMB9, PSMC3, PSMC4, PSMC5, PSMC6, PSMD12, PSMD4, PSMD7, PSMD8, PSME1, PSME2, PSME4*
Positive regulation of defense response	GO	3.07	7	23	*ADORA3, COLEC12, CTSS, HPX,* ***IL12B****,* ***IL1B****, IL6, IRAK2,* ***LTA****,* ***LTF****, MAPKAPK3, MEF2C, POLR3C, RIPK2, SERPINE1, STAT5B, TICAM2, TLR10, TLR2, TLR4, TLR6, TMEM173,* ***TNF***
Telomere maintenance	R	3.07	3	17	*HIST1H2BA, HIST1H2BB, HIST1H2BJ, HIST1H2BL, HIST1H2BN, HIST2H2AA4, HIST2H2AC, HIST2H2BE, POLD2, POT1, PRIM2,* ***RFC3****, RPA1, RPA3, TERF1, TERF2, TINF2*
Proteasome complex	GO	3.06	9	18	*PSMA5, PSMA6, PSMB10, PSMB5, PSMB8, PSMB9, PSMC4, PSMC5, PSMC6, PSMD12, PSMD4, PSMD7, PSMD8, PSME1, PSME2, PSME4, RAD23B, UCHL5*
Protein dimerization activity	GO	3.06	9	79	*ABAT, ABCG2, ADRA1A, ADRB3,* ***APOE****, ARNT, ASCL2, CAMK2B, CAT, CEBPG, CHMP4A, CHRNA7, CISD2, COQ9, CPQ, CRYM, CYBA, CYBB, DGAT2, DLK2, DNM1L, DPYD,* ***E2F8****, EBF2, ECE1, FBLN5, FDX1,* ***FOXP1****, GABPB2, GALE, GATA3, GDF6, GNPTG, GSTA4, H2AFJ, H2AFV, H2B, H4, HIST1H2BB, HIST1H2BN, HIST2H2AC, HMGCL, ID3,* ***IL12B****, IRAK2, ITGA3, IZUMO3, LHPP, LOC504599, MEF2C, MPP7,* ***MSH2****, MVD, MYC, MYOD1, MYOM1, NEUROD6, NR2F2, ODC1, PEX11B,* ***PKD2****, POLR1C, POLR2C, PPP3CA, PRDM6, PSMD7, S100A10, SLC39A13, SLC51A, STAT5B, STK4, TAF13, TCF23, TFEC, TMEM173, TNNC1, VEGFA, VPS25, XBP1*

Regulation of ornithine decarboxylase ODC	R	3.06	1	20	*AZIN1, NQO1, ODC1, PSMA5, PSMA6, PSMB10, PSMB5, PSMB8, PSMB9, PSMC3, PSMC4, PSMC5, PSMC6, PSMD12, PSMD4, PSMD7, PSMD8, PSME1, PSME2, PSME4*
Single organism biosynthetic process	GO	3.06	11	144	*ADAL, ADAMTS2, ADCY1, ADM, ADRB3, AGPAT1, AGPAT4, AJUBA, AK4, AK5, ALAS1, ALDH7A1, ALDH8A1, ANG, APIP, APOA2, APOC2,* ***APOE****, ASIP, ASNS, ASNSD1, ASS1, ATP5S, BDH2, BRCA1, CDH3, CHPT1, COA3, COQ2, COQ4, COQ6, COQ9, CREBL2, CTNS, CYB5R1, CYP11A1, CYP11B1, CYP17A1, CYP21, DEGS1, DEGS2, DGAT2, DHCR7, DHPS, DPH3, DPM3, DPYD, DRD1, DRD5,* ***E2F8****, EDNRA, ELOVL5, ENOPH1, EXT2, FAM213B, FASN, FDFT1, FDX1, FECH, FITM1, FXN, G6PC, G6PC3, GATC, GLCE, GNG7, GNPAT, GOT2, GPLD1, GUCY2F, HMGCL, HMGCS2, HOGA1, HTR2A, IFNG, IGF2,* ***IL1B****, IL6, IMPDH1, IP6K3, ISPD, LEP, LOC782922, LSS, MGST2, MMAB, MRPL44, MRPS18C, MTG1, MTHFD1L, MTRR, MVD, MVK, NADSYN1, NDST2, NME7, NOS3, OAS2, OAT, ODC1, OPRM1, PC, PEMT, PHGDH, PHKG2, PIGK, PIGY, PIK3CA, PLIN5, PLP1, PMEL, PNPO, PPARGC1A, PTCD3, PTDSS1, PTGES, PTGES2, PTGIS, PTGS1, PTH, RAPGEF2, RDH10, RDH8, SCD5, SDR42E1, SERAC1, SERINC1, SIRT4, SLC44A4, SMPD1, SNCA, SOD1, SPTLC1, SRD5A1, ST6GALNAC6, TAMM41, TBPL1, TFAM,* ***TGFB1****, TM7SF2, TMEM14C,* ***TNF****, TYMS, UMPS*
Cytoplasmic membrane bounded vesicle	GO	3.05	10	83	*AP1S1, AP2A2, AP3B1,* ***ARRB1****, ATP1B3, ATP6V0A1, ATP6V1E2, ATP8A1, BDNF, CARTPT, CCT4, CHGA, CHMP4A, CHP1, CLIC5, CLK3, CLU, COMMD1, COPG2, COPS4, CTSB, CTSH, CYB561, DMTN, DNM1L, DTNBP1, FASN, FLOT1, GCHFR, GLIPR1L1, HAX1, HCRT, HSP90B1, HTR2A,* ***IL1B****, ITPR2, ITPR3,* ***LTF****, NAP1L1, OVGP1, PACSIN1, PAM, PCSK1, PDIA3, PIFO, PLA1A, POMC, PPIB, RAB3C, RAB5C, RAB7A, RAB8B, SAYSD1, SCAP, SCGN, SEC23A, SERPINA5, SLC1A4, SLC6A17, SNCA, SND1, SNX17, SNX24, SPACA4, SPERT, SPG21, SPX,*

					STX19, STX1A, SV2A, SVOP, SYT1, TBC1D7, TLR6, TMEM198, TMEM30A, TRIM9, USO1, VOPP1, VPS51, YIPF5, YKT6, YWHAZ
Phosphatase regulator activity & protein phosphatase regulator activity	GO	3.04	1	8	*ENSA, PPP1R35, PPP1R37, PPP2R2B, PPP2R5E, RCAN1, RCAN2, RCAN3*
Regulation of secretion	GO	3.03	1	69	*ADORA1, ADORA3, ANG, APOA2,* ***ARRB1****, CARTPT, CDK5, CHGA, CHIA, CSN2, CYBA, DNM1L, DPH3, DRD2, DTNBP1, ENSA, FCER1G, GATA3, GH1, GHRL, GLRA1, GLUD1, GOLPH3L, GPLD1, GPR68, HTR2A, IFNAR1, IFNG, IL10,* ***IL1B****, IL6, INHBB, INS, IP6K3, LAMP1, LGR4, MEF2C, NLRP3,* ***NOD2****, NR1H3, OPRM1, PFN2, PLA2R1, PLCB1, POMC, PPID, PYCARD, RAB15, RAB27B, RAB3C, RABGEF1, RAP1A, RHBDF1, SCIN, SERGEF, SIRT4, SNCA, SPX, STX1A, SYT1,* ***TGFB1****,* ***TGFB2****, TLR10, TLR2, TLR4, TLR6,* ***TNF****, TRAF6, XBP1*
Defense response to other organism	GO	3.03	7	50	*APOA2, CAMP, CATHL1, CATHL3, CATHL4, CHGA, CXCL10, CXCL9, DEFB1, DEFB10, DEFB4A, DEFB7, ELMOD2, FCER1G, H2B, IFNAR1, IFNG, IFNT3,* ***IL12B****, IL15, IL6, IRF5,* ***ISG15****, LAP, LPO,* ***LTA****,* ***LTF****, LYZ1, LYZ2, LYZ3, MR1, NCF1, OAS2, PENK, PLAC8, POLR3A, POLR3C, POLR3K,* ***RBPJ****, ROMO1, SEH1L, SERPINE1, TICAM1, TLR4, TMEM173,* ***TNF****, TNFAIP8, TRIM11, TRIM38, TRIM56*
Steroid metabolic process	GO	3.03	5	33	*AFP, AGTR1, APOA2,* ***APOE****, CAT, CYB5R1, CYP11A1, CYP11B1, CYP17A1, CYP21, DHCR7, FDFT1, FDX1, FECH, G6PC, HMGCS2, IFNG,* ***IL1B****, LSS, MBTPS2, MVD, MVK, NPC2, RDH8, SCAP, SDR42E1, SOD1, SRD5A1, STAT5B, SULT1B1, SULT1E1, TM7SF2,* ***TNF***
Cross presentation of soluble exogenous antigens endosomes	R	3.02	10	19	*FCGR1A, MRC2, PSMA5, PSMA6, PSMB10, PSMB5, PSMB8, PSMB9, PSMC3, PSMC4, PSMC5, PSMC6, PSMD12, PSMD4, PSMD7, PSMD8, PSME1, PSME2, PSME4*

Endoplasmic reticulum phagosome pathway	R	3.01	6	19	*B2M, PDIA3, PSMA5, PSMA6, PSMB10, PSMB5, PSMB8, PSMB9, PSMC3, PSMC5, PSMC6, PSMD12, PSMD4, PSMD7, PSMD8, PSME1, PSME2, PSME4, TAP1*

[1]Databases: Gene Ontology (GO) http://www.geneontology.com, Kyoto Enclyopedia of Genes and Genomes (KEGG) http://www.genome.jp.kegg, Reactome (R), Panther http://www.pantherdb.org. [2]Normalized enrichment score (NES). 3Functional classifications: regulatory (1), cellular differentiation/development (2), DNA/RNA regulation (3), immune function (4), metabolism (5), pathways (6), cellular responses (7), enzyme activity (8), proteasomes (9), vesicles (10), biosynthetic processes (11), and cell signaling (12). [4]Total number of leading-edge genes (LEGs) within the gene set. [5]Leading-edge genes enriched for bovine respiratory disease from each gene set. Bolded genes were identified as leading-edge genes in both pre- and post-weaned BRD groups. Underlined genes were found in approximately half of the gene sets for each respective population.

Supplemental Table S4. Genes associated with bovine respiratory disease that are shared in the current and previous bovine respiratory disease studies.

Study	# Genes[1]	Shared Genes[2]
Neibergs et al., 2014	4	*AZIN1, BAALC, TRNAC-ACA, TRNAG-CCC*
Tizioto et al., 2015	451	*A2M, AAK1, ABHD12, ABT1, ACADS, ADAMTS2, ADCY2, ADM, ADORA3, ADRB3, AGMO, AGTR1, AHCY, AIF1, AK8, ALB, ALDH6A1, ALMS1, ALPL, ANAPC1, ANG, APC, APIP, APOE, ARHGAP15, ARL2, ARL3, ASCC3, ASCL2, ASIP, ASNS, ASPH, ASS1, ATOH8, ATP1A1, ATP1B3, ATP6V1F, ATP8A1, ATR, BCAM, BLVRB, BMI1, BRCA1, BUB1, C1H3orf70, C2, C4A, C4BPA, C6, CAMTA1, CAPN2, CAPN3, CAV1, CBLC, CCL11, CCND2, CCRL2, CCT4, CD81, CDC14A, CDC25C, CDH3, CDK13, CDK4, CDK5RAP3, CENPN, CEP192, CFB, CFH, CHGA, CHID1, CISH, CLASP1, CLCN7, CLIC1, CLIC4, CLIC5, CLPB, CLU, CNPY3, COL4A3BP, COLEC12, CPT1B, CREM, CRYAB, CSNK1A1, CTHRC1, CTSB, CTSC, CTSH, CTSK, CX3CR1, CXCL10, CXCL16, CXCL9, CYBA, DAP, DDAH2, DDIT3, DEFB10, DEFB4A, DEFB7, DENND4A, DGAT2, DIDO1, DMBT1, DNA2, DNAH7, DNMT1, DPM3, DPYD, DTNBP1, DYRK1A, E2F2, E2F8, ECSCR, EDNRA, EDNRB, EEF1A2, EFNA1, EIF4EBP1, ELMOD2, ELOVL5, ENTPD1, ENTPD6, ENTPD8, ETFDH, ETV1, F3, FABP7, FAH, FAP, FASN, FBLN5, FCER1G, FCGR1A, FKBP5, FLOT1, FN1, FOLR2, FZD6, GABPB2, GADD45G, GALE, GAPDH, GAPVD1, GATA3, GBA, GCHFR, GEMIN7, GINS1, GINS2, GINS4, GLB1, GLUD1, GMPR, GNAO1, GNAT2, GOT1L1, GPNMB, GPX1, GSTA2, GSTA4, GSTP1, GTF2H2, GUCY2C, H2AFJ, H2AFV, H4, HAGH, HAUS2, HAX1, HBS1L, HDLBP, HIST1H2BN, HIST2H2BE, HOMER1, HPGD, HRH1, HSP90B1, HTRA3, ID3, IFNAR1, IFNG, IGF2, IGF2BP3, IL10, IL12B, IL18BP, IL1B, IL1RN, IL6, IMPDH1, INHBB, INPPL1, IRF5, ISG15, ITGA3, ITIH4, ITPR3, KANK2, KCNIP3, KCNK1, KCNMA1, KIAA1324L, KIF13B, KIF18A, KIF23, KITLG, LAMP1, LAMTOR1, LEP, LETM1, LGR4, LHPP, LIN54, LOC100848407, LOC100848958, LOC782922, LOC784768, LSS, LTA, LTC4S, LTF, LYST, LYVE1, LYZ2, MAGOH, MAN2B1, MAP1LC3A, MAPK1, MAPKAPK3, MCEE, MEF2C, MEST, MIS18BP1, MMS22L, MOV10, MPP7, MPV17, MRC2, MRPL44, MSH2, MTHFD1L, MVK, MYL6, MYO1D, NAP1L1, NARS, NCF1, NEDD1, NENF, NFIB, NFKBIB, NME3, NME6, NOS3, NPC2, NPY, NR1H3, NR2F2, NR4A1, NUDT12, NUSAP1,*

		OAS2, OAT, ODC1, OLFML2A, OSR1, PACSIN1, PAM, PARG, PC, PCNT, PDE1C, PDE3A, PDE4D, PDIA3, PFN2, PHGDH, PIGK, PIK3CA, PIK3R1, PLA1A, PLA2R1, PLAC8, PLCB1, POLD4, POLE2, POLR2F, POLR2K, POLR3A, POLR3K, POMC, PPA1, PPIB, PPID, PPP1CB, PPP1R35, PPP2R2B, PPP2R5A, PPP2R5C, PPP2R5E, PPP3CA, PRDM6, PRKAR2A, PRKAR2B, PRKCB, PRKD1, PRKRIP1, PRMT5, PRNP, PROS1, PSMA5, PSMA6, PSMB10, PSMB5, PSMB8, PSMB9, PSMC3, PSMC5, PSMD12, PSMD4, PSMD8, PSME1, PSME2, PTGDR, PTGES, PTGES2, PTGIS, PTGS1, PTPN5, PYCARD, RAB27B, RAB30, RAB5C, RAB6B, RAB8B, RABGEF1, RAN, RAPGEF2, RAPGEF5, RASA1, RBBP8, RBM8A, RBMS1, RBPJ, RCAN3, RCE1, REC8, REV3L, RHBDF1, RHOB, RIPK3, ROMO1, RRM2, S100A10, SAR1A, SARS, SCARA5, SCD5, SCHIP1, SCIN, SEMA3A, SEMA3C, SEMA3D, SENP7, SERAC1, SERPINA5, SHMT2, SKAP1, SLC16A1, SLC1A4, SLC39A13, SLC6A17, SLU7, SMAD1, SMARCAD1, SMC3, SMPD1, SMPDL3A, SNCA, SND1, SNRPC, SNRPE, SNX11, SNX13, SNX24, SOCS2, SOCS5, SPCS1, SPCS3, SPG21, SPTY2D1, SSNA1, ST6GALNAC6, STAG1, STAG2, STAG3, STK38, STK4, STRBP, STX1A, SYNDIG1L, SYNE1, SYNE2, TALDO1, TCN2, TFAM, TFEC, TGFB1, THEM4, TJP2, TLR10, TLR2, TLR4, TLR6, TM9SF1, TMEM173, TMEM44, TMEM51, TMEM86A, TNF, TNFAIP8, TOLLIP, TRA2A, TREX1, TRIB2, TRIM21, TRIM38, TUBG1, TXN2, UCHL3, UMPS, UPB1, USP1, USP13, USP15, USP34, VEGFA, VOPP1, VPS13B, WARS, WEE1, WWP1, XBP1, XDH, XPO1, YIPF5, YWHAZ, ZW10
Kiser et al., 2017	3	*HPX, IP6K3, ITPR3*
Neupane et al., 2018	98	*A2M, ACADS, ACADSB, ADORA3, ALDH8A1, ANAPC15, APOC2, APOE, ARRB1, ASNS, ASPH, ATP1B3, C6, CACNB2, CACNG3, CAPN3, CAV1, CHRNA7, CLU, CPT1B, CROT, CRYAB, DAP, DEGS1, DNMT1, DPP6, DPYD, DRD2, EDNRB, EIF4EBP1, ELOVL5, ENOPH1, ENTPD5, ESR1, FASN, FHIT, FN1, GLRA1, GPLD1, HK1, HOGA1, HPGD, HSD17B12, HTR2A, IGF1R, IL1B, IL6, IRAK2, ITGA3, ITIH1, KCNH1, KCNIP3, KCNJ3, KCNMA1, KCNQ3, LCMT1, LEP, LGR4, LONP2, LTC4S, MEN1, MGST2, MTHFD1L, NLRP3, NR1H3, NR2F2, PBLD, PLCB1, PLK1, PPARGC1A, PRKAR2A, PRKRIP1, PRNP, PSEN1, PSME4, PTGIS, RAD23B, SCD5, SERPINA5, SERPINC1, SIRT4, SLC1A3, SNCA, SNTA1, SOCS2, SOCS5, STK38, TNF, TNFAIP8, TRIB3, TRIP12, USP13, USP20, USP4, XBP1*

Quick et al., 2020	1	*TRIM21*
Hasankhani et al., 2021	312	*ABAT, ABCC1, ABHD12, ADAMTS2, ADCY3, ADORA3, AHCY, AIF1, AK4, AK7, AKIRIN2, ALDH7A1, ALMS1, ALPL, AMPD2, ANAPC15, AP2A2, ARL2, ARNT, ATG3, ATP1B3, ATP6V0A1, ATP6V1A, ATP8A2, BMI1, BRCA1, BUB1, BUB3, C1H3orf70, CACNB2, CAPN2, CAPN3, CAPNS1, CAT, CATHL3, CBLC, CCRL2, CD81, CDC14A, CDC25C, CDCA7L, CDH3, CENPA, CENPN, CFB, CFL1, CHMP4A, CHP1, CISH, CLASP1, CLCN7, CLIC4, CLU, COQ2, COQ4, COQ6, CPT1B, CREBL2, CREM, CROT, CRTC3, CRYAB, CTSB, CTSC, CTSH, CTSK, CTSS, CX3CR1, CYB5R1, CYBA, DARS2, DDAH2, DDIT3, DDX39B, DEFB10, DEFB4A, DEFB7, DGAT2, DNA2, DNMT1, DPYD, DYNLRB1, E2F2, E2F3, E2F8, ECE1, ECSCR, EEF2, EFNA1, EHD1, ENPP2, ENTPD1, ERCC6L2, ESR1, EXOC4, FASN, FCER1G, FCGR1A, FLOT1, FN1, FYN, GABPB2, GAPDH, GAPDHS, GATA3, GATC, GBA, GCHFR, GEMIN7, GGT7, GINS1, GINS3, GINS4, GNAO1, GNG7, GNPTG, GOLPH3L, GOT2, GPX1, GSTP1, GTF2F2, HAUS2, HDLBP, HMGCL, HOGA1, HSP90B1, IFNAR1, IFNAR2, IL10, IL12B, IL15, IL18BP, IL1B, IL1RN, ISG15, ITGA10, ITPR3, KCP, KLC4, LAMP1, LAMTOR1, LAMTOR5, LARGE1, LSM8, LTA, LTF, MAP1LC3A, MAPK1, MAPKAPK3, MBTPS2, MCM3, MEF2C, MGST2, MIS18A, MIS18BP1, MLH1, MMS22L, MOV10, MPP7, MR1, MRC2, MSH2, MTHFD1L, MTRR, MYC, NADSYN1, NCF1, NDEL1, NDOR1, NECTIN2, NEDD1, NFKBIZ, NOD2, NPC2, NPM1, NR1H3, NR4A1, NRROS, NSD2, NUDT3, NUSAP1, OAS2, ORC3, OXR1, PACSIN1, PAFAH1B2, PAM, PC, PCNT, PHKG2, PIAS3, PIK3CA, PKD2, PLCB1, PLK1, POLA2, POLD1, POLD2, POLD3, POLD4, POT1, PPP1R35, PPP2R5A, PPP2R5C, PRKAR2B, PRKRIP1, PSEN1, PSMA6, PSMB8, PSMB9, PSMC3, PTCD3, PTGDR, PTPN5, PTTG1, PYCARD, RAB5C, RAB7A, RAC1, RAD51, RAN, RBBP8, RBPJ, RCAN3, REC8, REEP2, RHOBTB1, RIPK1, RIPK3, RPA1, RPA3, RRM2, RSRC1, S100A10, S1PR1, SCAP, SEMA3C, SERAC1, SHMT2, SLC16A1, SLC25A5, SLC8A1, SLU7, SMAD1, SMARCAD1, SMPD1, SMPDL3A, SMS, SNRPE, SNTA1, SNX11, SNX13, SNX24, SPCS1, SPCS3, SPG21, SPTLC1, SPTY2D1, SRSF6, SRSF7, SSNA1, STAG1, STAG2, STAT5B, STRBPSV2A, SYNE2, TALDO1, TAMM41, TAP1, TARBP1, TBC1D14, TCN2, TFAM, TFDP1, TFEC, TG, TICAM2, TIPIN, TLR10, TLR2, TLR4, TM7SF2, TMEM198, TMEM30A, TMEM51, TNF, TNFAIP8, TNFAIP8L2, TRA2A, TRAF3IP2, TREX1, TRIB2, TRIB3, TRIM21, TRPC6, TUBB, TUBGCP6, TYMS,*

		UCKL1, UPB1, USP1, USP10, USP20, USP4, WEE1, WWP1, XBP1, XDH, YWHAZ, ZRANB1, ZW10
Cao et al., 2024	3	*LTA, NOD2, TLR2*
Strillacci et al., 2025	3	*ATP6V1A, MPC1, PLCB1*

[1]The number of genes that were shared. [2]List of shared genes previously identified as associated with, differentially expressed for, or a leading-edge gene for BRD.

Supplemental Table S5. Gene sets enriched with bovine respiratory disease that are shared in the current and previous bovine respiratory disease studies.

Study	# Genes Sets[1]	Gene Set Names[2]
Hasankhani et al., 2021	5	Cellular response to oxidative stress (GO:0034599)<br>Cellular response to oxygen-containing compound (GO:1901701)<br>Negative regulation of kinase activity (GO:0033673)<br>Negative regulation of protein kinase activity (GO:0006469)<br>Positive regulation of defense response (GO:0031349)

[1]The number of gene sets that were shared. [2]List of shared gene sets previously identified as associated with BRD.
